# Amorphous porous organic polymers containing main group elements

**DOI:** 10.1038/s42004-023-01063-5

**Published:** 2023-12-11

**Authors:** Zhikai Zhang, Zhaoxin Liu, Cece Xue, Hongyi Chen, Xue Han, Yi Ren

**Affiliations:** School of Physical Science and Technology, ShanghaiTech University, Shanghai, 201210 China

**Keywords:** Materials chemistry, Inorganic chemistry, Porous materials

## Abstract

Amorphous porous organic polymers (*a*POPs) are a type of highly crosslinked polymers. These polymers are generally constructed from rigid organic building blocks, which have become an important subclass of POPs with diverse applications. In the early stage of development, a wide range of carbon-based building blocks and network forming chemistry afforded a large library of *a*POPs with rich structures and properties. Recently, implanting main group elements with diverse geometric structures and electronic configurations into *a*POPs has proven to be a useful tool to fine-tune the structures and properties of these polymers. Herein, we outline the recent advances in the field of main group (MG)-*a*POPs where main-group elements either played unique roles in tuning the structures and properties of MG-*a*POPs, or offered new strategies in the synthesis of MG-*a*POPs. Furthermore, this Review discusses various challenges remaining in the field from the perspectives of synthetic strategies and characterization techniques, and presents some specific studies that may potentially address the challenges.

## Abstract (web summary)

Incorporating main group elements into amorphous porous organic polymers has enabled the fine tuning of the structures and properties of these materials. Here, the authors review studies in which the geometric structures and electronic properties of main group elements have influenced material structures and properties, and whereby their incorporation has enabled new strategies to synthesize such materials.

## Introduction

Porous organic polymers (POPs) have gained significant attention in the diverse applications, such as gas storage/separation, heterogeneous catalysis, energy storage, sensing, and other optoelectronic devices^1–5^. Different from zeolites and metal-organic frameworks containing metal ions, POPs are a subclass of porous materials containing only organic building blocks. Generally, POPs can be grouped into crystalline (*c*)POPs, such as covalent organic frameworks (COFs)^4^, covalent triazine frameworks (CTFs)^6,7^, and amorphous (*a*)POPs, such as conjugated microporous polymers (CMPs)^2,8,9^, porous aromatic frameworks (PAFs)^3,10–12^, hypercrosslinked polymers (HCPs)^13–15^, and polymers of intrinsic microporosity (PIMs)^16–18^.

As the first report of *c*POPs, Yaghi and coworkers reported the synthesis and porous structures of COFs in 2005^19^, in which the highly ordered and tunable porous structures endowed the *c*POPs with unprecedented opportunities in wide applications^4^. As the twin sister of *c*POPs, research of *a*POPs has witnessed a long historical development. In late 1960s, Davankov and coworkers reported the first example of HCPs synthesized by the Friedel-Crafts catalyzed self-condensation of p-dichloroxylene. The HCPs exhibited the apparent Brunauer-Emmett-Teller (BET) surface areas up to 1106 m^2^ g^-1^ ^20^. Using the similar synthetic method, Tan and coworkers recently reported a new series of HCPs with layered microporous structures, which exhibited the BET surface area as high as 3002 m^2^ g^-1^ ^21^. Being a unique member of *a*POPs, the first kind of PIMs was reported by McKeown and Budd in 2004^16^. The porous character of these polymers is originated from the high rigid structures and the contorted shapes in the linear polymeric backbone, in which the polymers cannot pack efficiently. The solution processable character makes PIMs highly distinctive from other *a*POPs.

Later on, the rich and well-developed synthetic protocols further accelerated the development of *a*POPs. For example, transition-metal catalyzed carbon–carbon (C–C) cross-coupling reactions, such as Sonagashara-Hagishara^8,9^, Suzuki-Miyaura^22–24^, Yamamoto ^10,25–27^, Heck^28,29^, etc. became the widely used synthetic protocols to construct *a*POPs. Leveraging on the well-documented C–C coupling reactions, a mix and polymerize strategy has become very popular and appealing in the field of *a*POPs. In 2007, Cooper and coworkers reported the first example of CMPs with various π-conjugated structures^8^. Although some optimizations were required, Sonagashara-Hagishara coupling reaction generally afforded the CMPs with high surface areas and microporous structures^8,30^. Later, the tunable porous structures and optoelectronic properties were realized by carefully choosing monomers with different chemical and electronic nature^8,30,31^. In 2009, using Yamamoto cross-coupling reactions, Ben and coworkers reported the first example of PAF containing tetrahedral tetrakisphenyl methane (TPM) building block, The PAF exhibited an extremely high BET surface area of 5640 m^2^ g^-1^^10^. The high surface-area character is highly beneficial for the applications in gas storage and gas separation.

Along with maturing of synthetic protocols, research of exploring diverse functions has been blooming in the field of *a*POPs^1–3,5,9,15,32^. The structures and properties of *a*POPs are highly dictated by the chemical structures and porous structures. Gas storage (H_2_, CH_4_, and CO_2_) and separation have been the largest area of study for designing new *a*POPs with the high surface area^33–35^. Tunability of the porous diameters and compositions (electron rich or electron poor) offered *a*POPs with the improved adsorption capacity and selectivity^12,26,36^. Recently, extensive usages of organic π-conjugated building blocks in the rigid polymeric structures further extended *a*POPs in the light-emitting related applications^27,37^. The light-emitting properties were highly tunable by modulating the chemical structures of the building blocks. *a*POPs with light-emitting characters were further applied for the detection of various chemicals by monitoring the changes of light-absorption and photoluminescence^38–41^. The large open sites also allowed the strong interactions of *a*POPs with the various chemicals, thus enhancing the signal sensitivities for the detections. Furthermore, the high chemical and topological stability provided a good platform for *a*POPs in the application of heterogeneous catalysis^5,42–44^. Reactants were able to easily access the open porous structures and reach to the catalytic sites on the backbones. Besides, *a*POPs with various photo-active building blocks became the promising candidates as the photoredox catalysts for chemical conversions^45,46^ and water splitting^47–49^. In addition to the applications mentioned above, *a*POPs also found interests in energy storage^50,51^, biosensing^52,53^, drug delivery^54^, antibacterial^55,56^, and phototherapy^57^.

As a special subgroup of *a*POPs, main group element (such as boron, nitrogen, phosphorus, silicon, sulfur, etc.) containing (MG)-*a*POPs slowly draw attention in recent years^38–40,58–61^. The rich electronic configurations and geometries of the main group elements provided a new dimension to fine-tune the structures, properties and functions of *a*POPs. For example, incorporating trivalent B-center with an empty p-orbital endowed *a*POPs with excellent toxic anions and chemicals (fluoride, cyanide anoins, amines, etc.) sensing properties^38,39^. In presence of Lewis base N-center, *a*POPs exhibited the enhanced Lewis basicidity where the polymers exhibited better Lewis acid gas (CO_2,_ SO_2_, etc.) takeup^62–64^. The strong interactions between S-center and lithium ion allowed *a*POPs to show the promising redox characteristics in lithium-ion batteries^51^. Thus, these unusual characteristics and applications that are not easily accessible to the traditional pure-carbon based *a*POPs can be realized in MG-*a*POPs.

In this review, we present the recent developments of MG-*a*POPs. The review does not aim to comprehensively cover the whole field of MG-*a*POPs, but rather a personal selection of recent papers where the main-group elements either play important roles in tuning the structures and properties, and/or offering new strategies in the synthesis of the MG-*a*POPs. This review organizes MG-*a*POPs into Group-13 *a*POPs, Group-14 *a*POPs, Group-15 *a*POPs, and Group-16 *a*POPs, in which the specific main-group elements are involved. With the disussions of recent developments in MG-*a*POPs, the review seeks to clarify the challenges remaining in the field, and propose alternative approaches potentially to address the challenges.

### Group 13 containing amorphous POPs

Compared with other elements in group 13, boron element is one of the most extensively studied in the field of MG-*a*POPs. Having an empty p-orbital, trivalent B-center exhibits strong Lewis acid character and electron-accepting character. Therefore, doping trivalent B-center into small molecules and macromolecules endowed the systems with excellent toxic chemicals sensing properties and electron accepting/transporting properties^51,65,66^. Leveraging on extensive research on B-based small molecules and macromolecules in the literature, a number of new B-*a*POPs were rationally designed and constructed in recent studies.

**Fig. 1:**
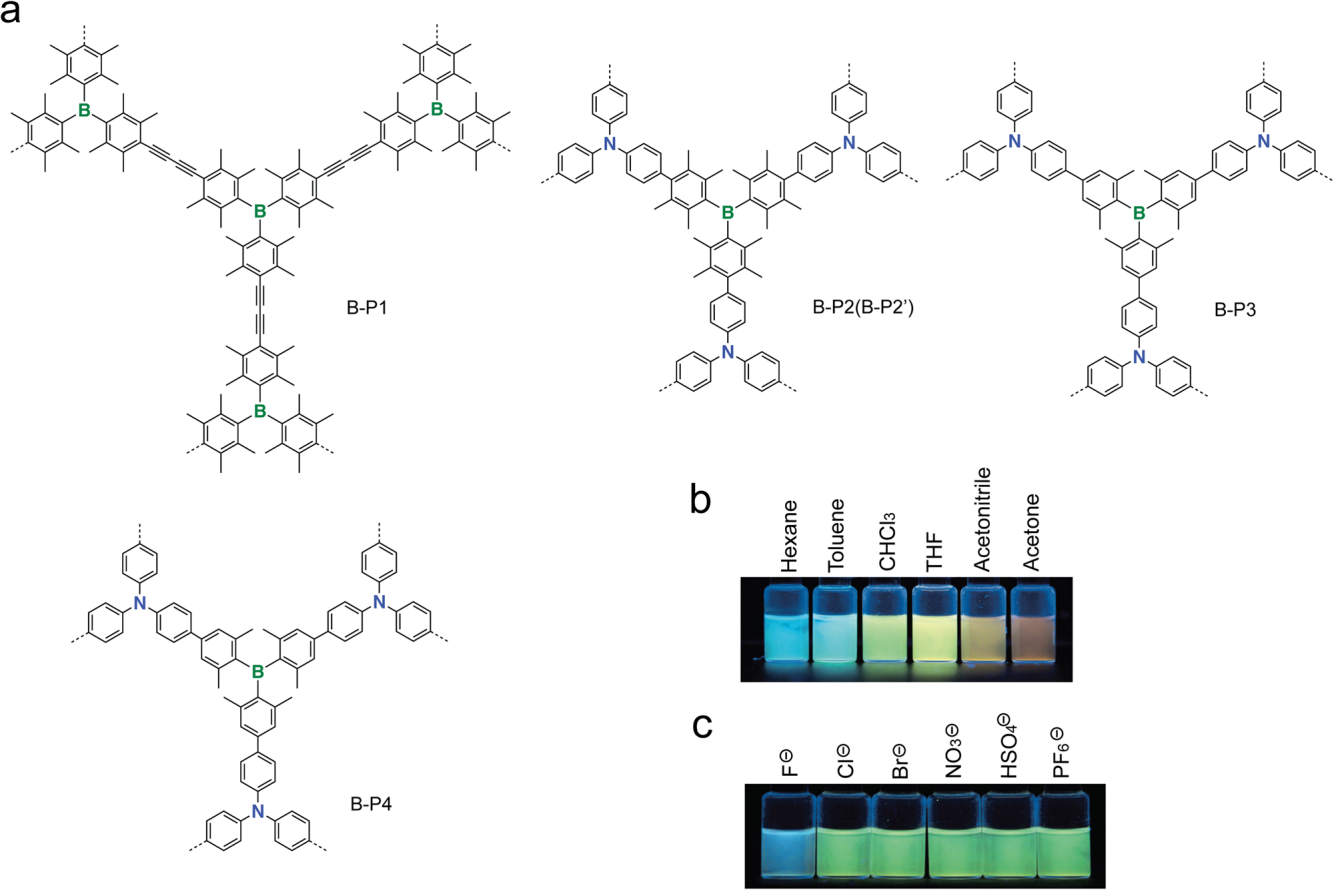
B-aPOPs with trivalent B-center. **a** Chemical structure of B-*a*POPs with trivalent B-center in the selected literature^38,40,96^. **b** Solvent dependent emission and (**c**) anion response emission of B-P3 (adapted with permission from ref. ^96^. © 2015 John Wiley and Sons).

Liu and coworkers reported the first example of B-*a*POPs (B-P1 and B-P2**)** containing triaryl B-building blocks (Fig. 1a)^38^. Similar to previous studies^65–67^, the triaryl B-building block contained multiple methyl groups to protect the B-center in Sonagashira-Hagihara polymerization. Solid-state ^11^B nuclear magnetic resonance spectroscopy (NMR) experiments confirmed the presence of trivalent B-center in the B-*a*POPs. B-P1 and B-P2 showed the BET surface areas of 815 m^2^ g^−1^ and 911 m^2^ g^−1^, respectively. Later, the same group also reported B-P3 that was synthesized by Suzuki-Miyaura coupling reaction^38^. These B-*a*POPs generally displayed strong photoluminescence in both the dispersed solution and the solid state. Due to the presence of donor (N-containing moiety)—acceptor (B-containing moiety) type structure, B-P2 and B-P3 exhibited the strong solvent dependent emission via intermolecular charge transfer (ICT) state (Fig. 1b). Their studies further uncovered that B-P3 showed a highly selective photoluminescence responsive property towards fluoride anion (Fig. 1c). Almost at the same time, Feng and coworkers reported similar B-*a*POPs (B-P2’ and B-P4 in Fig. 1a). B-P2’ and B-P4 exhibit very similar porosity characteristics and photophysical properties to these of B-P1,2,3^40^.

**Fig. 2:**
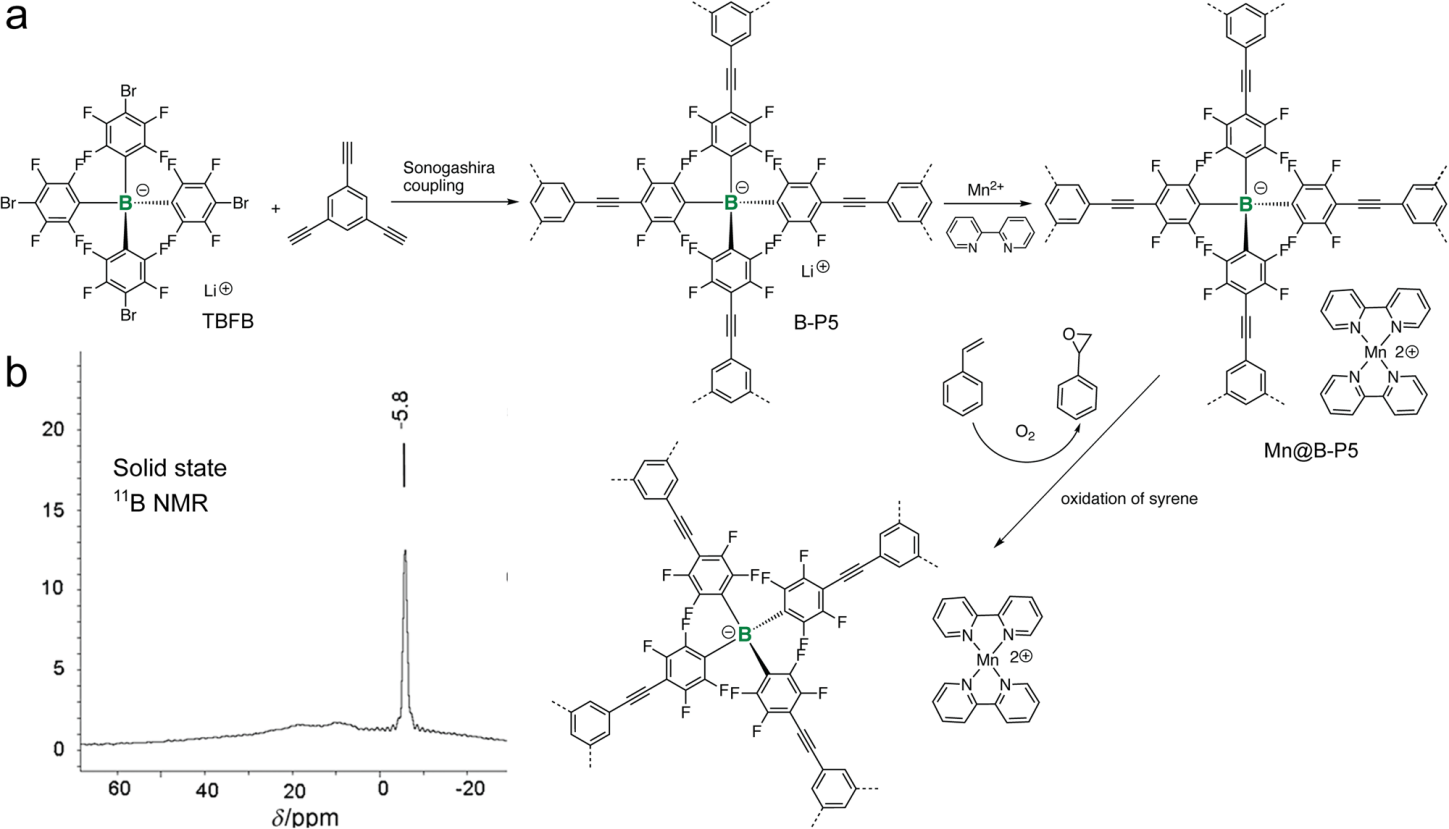
B-aPOPs with tetravalent anionic B-center. **a** Synthesis and post chemical modification of B-P5. **b** Solid state ^11^B NMR spectra of B-P5. (adapted with permission from ref. ^68^. © 2013 John Wiley and Sons).

In addition to trivalent B-building blocks, tetravalent B-building blocks were also recently explored in the synthesis of B-*a*POPs. Compare with trivalent B-center, tetravalent B-center generally exhibits better stabilities towards water and oxygen. In 2013, Thomas and coworkers reported a new type of B-*a*POP containing tetrahedral anionic B-center (TPFB in Fig. 2a)^68^. A strong signal at −5.8 ppm observed in solid state ^11^B NMR spectroscopy experiments suggested that B-P5 maintains a uniform tetravalent B-center in the polymeric backbones (Fig. 2b). Having TPFB unit structurally related to TPM, B-P5 exhibited a BET surface area of 890 m^2^ g^−1^. The microporous structure facilitated a facile ion-exchange between lithium ion and sodium ion in B-P5, which resembles to the silicon-aluminum exchange reaction in inorganic zeolites^69^. Further ion exchange with manganese bipyridine complexes allowed the converted Mn@B-P5 to be a promising catalyst for the oxidation of alkenes (Fig. 2a).

**Fig. 3:**
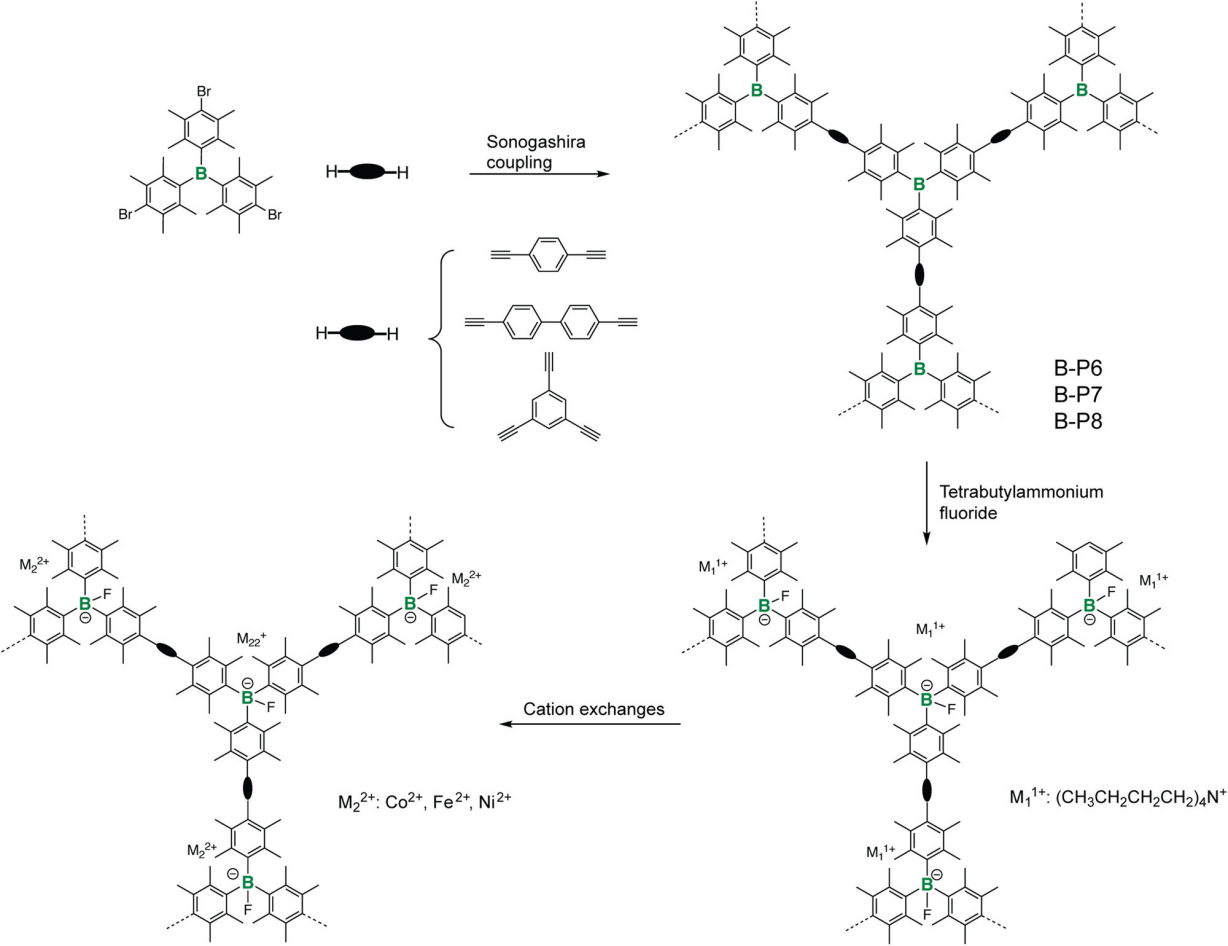
B-aPOPs with post-functionalized tetravalent B-center. Synthesis and post chemical modification of B-P6,7,8. (adapted with permission from ref. ^70^. © 2016 Royal Society of Chemistry).

Later, leveraging on strong binding between trivalent B-center and fluoride anion, Zhuang and coworkers used a post-polymerization chemical modification strategy to access tetravalent B-center (Fig. 3). B-*a*POPs (B-P6, B-P7, and B-P8) reacted with tetrabutylammonium fluoride as fluoride anion source^70^. Their studies revealed that most of the B-centers were successfully converted to the tetravalent B-centers by binding with fluoride anion. Presence of unreacted trivalent B-centers was correlated to the porous structure character in the different B-*a*POPs. They further applied ion-exchange reactions to the B-*a*POPs by reacting with various metal salts (cobalt acetate, nickel acetate, and iron nitrate). As a proof of concept, Co@BF-POP was used for the homocoupling reactions of aryl Grignard reagents where a distinct size selectivity was observed in the various porous structures. The study nicely demonstrated that the boron chemistry can be used as a ship in bottle technique in *a*POPs.

**Fig. 4:**
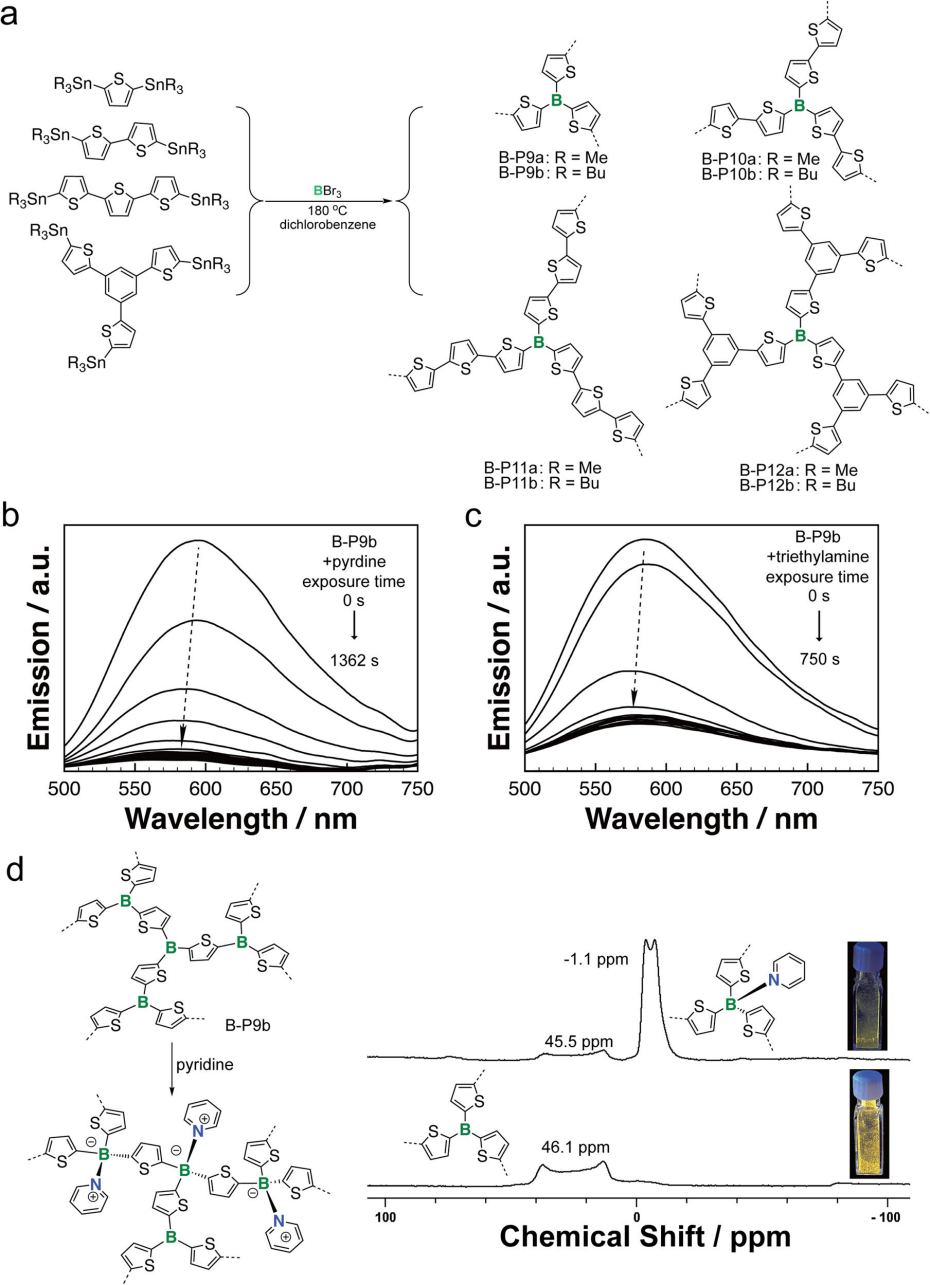
B-aPOPs with unprotected trivalent B-center. **a** Synthesis of B-P9a,b, B-P10a,b, B-P11a,b and B-P12a,b by B-Sn exchange reaction^39^. **b**, **c** Pyridine and trimethylamine sensing of B-P9b in the solid state. **d** Pyridine adsorption and solid-state ^11^B NMR spectra of B-P9b. (adapted with permission from ref. ^39^. © 2022 American Chemical Society).

So far, transition metal catalyzed C–C coupling reactions are the main synthetic protocol to access *a*POPs in the literature. Recently, Ren and coworkers explored boron-tin (B-Sn) exchange reaction as a new synthetic protocol to build B-*a*POPs (Fig. 4a, B-P9a,b, B-P10a,b, B-P11a,b and B-P12a,b)^39^. The new synthetic protocol allows the construction of the first examples of B*-a*POPs without sterically protected trivalent B-centers. The studies revealed that the B-*a*POPs maintained well-define chemical structures and microporous structures. Particularly, solid state ^11^B NMR spectroscopy experiments uncovered the highly uniform trivalent B-centers in the B-*a*POPs. They found that, compared with B-*a*POPs synthesized by trimethyltin oligothiophenes, B-*a*POPs synthesized by tributyltin oligothiophenes show better porous structures, such as high BET surface areas and high ratios of microporous structures. It was rationalized that the better solubility and low reactivity of tributyltin oligothiophenes were beneficial for constructing B-*a*POPs with the better porosity in the B-Sn exchange reaction.

Furthermore, B-*a*POPs with the unprotected B-center showed strong p-π* electronic coupling compared with the steric protected B-center, thus increasing Lewis acidity. With the enhanced Lewis acidity, these B-*a*POPs exhibited excellent amine and pyridine sensing and absorptivity (Fig. 4b, c). The B-*a*POPs showed reversible pyridine absorption properties. The pyridine absorptivity of B-P9b reached as high as 570 mg g^−1^. Solid state ^11^B NMR experiments of B-P9b futher confirmed the cooridination of the B-centers with pyridine molecules (Fig. 4d).

**Fig. 5:**
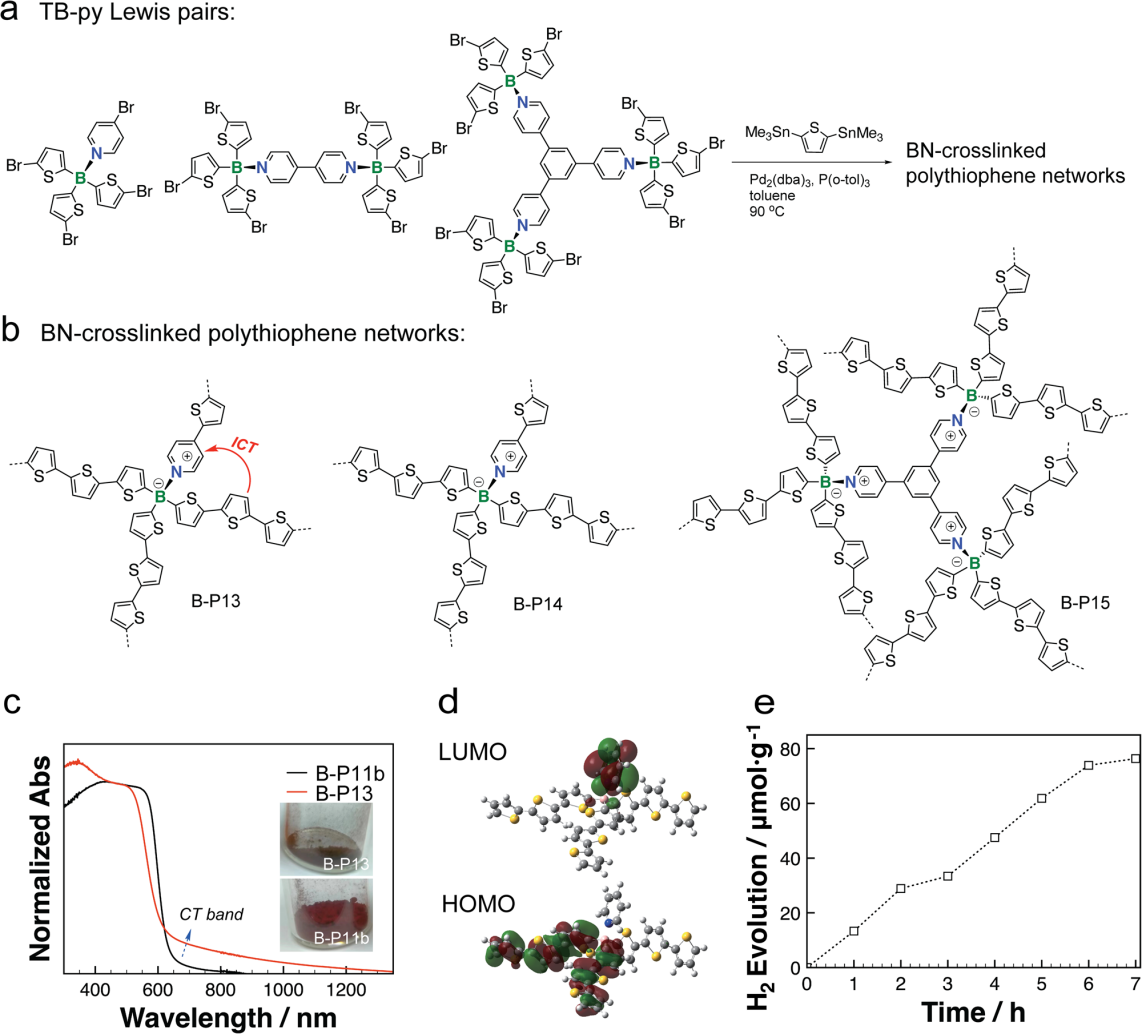
B-aPOPs with pyridine-coordinated B-center. **a** Chemical structure of TB-py Lewis pairs. **b** Chemical structure of BN-crosslinked polythiophene networks. **c** Solid state UV-vis diffuse reflectance spectra of B-P13 and B-P11b (inserted images of B-P13 and B-P11b powders). **d** Frontier molecular orbitals of model molecules at the level of TD-B3LYP/6-31+g(d). **e** Time course for photocatalytic H_2_ evolution of B-P13 under *λ* >400 nm light illumination. (adapted with permission from ref. ^71^ © 2022 American Chemical Society).

The high Lewis acidity of thienylborane towards various pyridine moieties (such as 4-bromopyridine, 4,4’-bipyridine, and 1,3,5-tri(pyridinyl)benzene) further inspired Ren and coworkers to design new thienylboran-pyridine (TB-py) Lewis pairs (Fig. 5a)^71^. BN-crosslinked polythiophene networks with the diverse topological TB-py Lewis pairs were constructed by using typical Stille type coupling reaction (Fig. 5a, b). The nonplanar TB-py Lewis pairs endowed the polymers with strong intramolecular charge separation characteristics (Fig. 5c, d). The theoretical studies of model molecules revealed that negligible molecular orbital coupling between the HOMO (thiophene moieties) and the LUMO (pyridine moieties) was responsible for the strong intramolecular charge separation characteristics (Fig. 5d). Furthermore, B-P13 is highly stable towards the high temperature and water, which showed promising photocatalytic hydrogen production properties in the preliminary studies (Fig. 5e). It needs to be noted that the BN-crosslinked polythiophene network is not the first examples of B-*a*POPs that were applied in the application of photocatalytic hydrogen production. For example, Pan’s group reported a new example of B-*a*POPs with trivalent B-center showing competitive hydrogen evolution rate (HER) as high as 1603 μmol h^−1^ g^−1^ under visible light^72^.

**Fig. 6:**
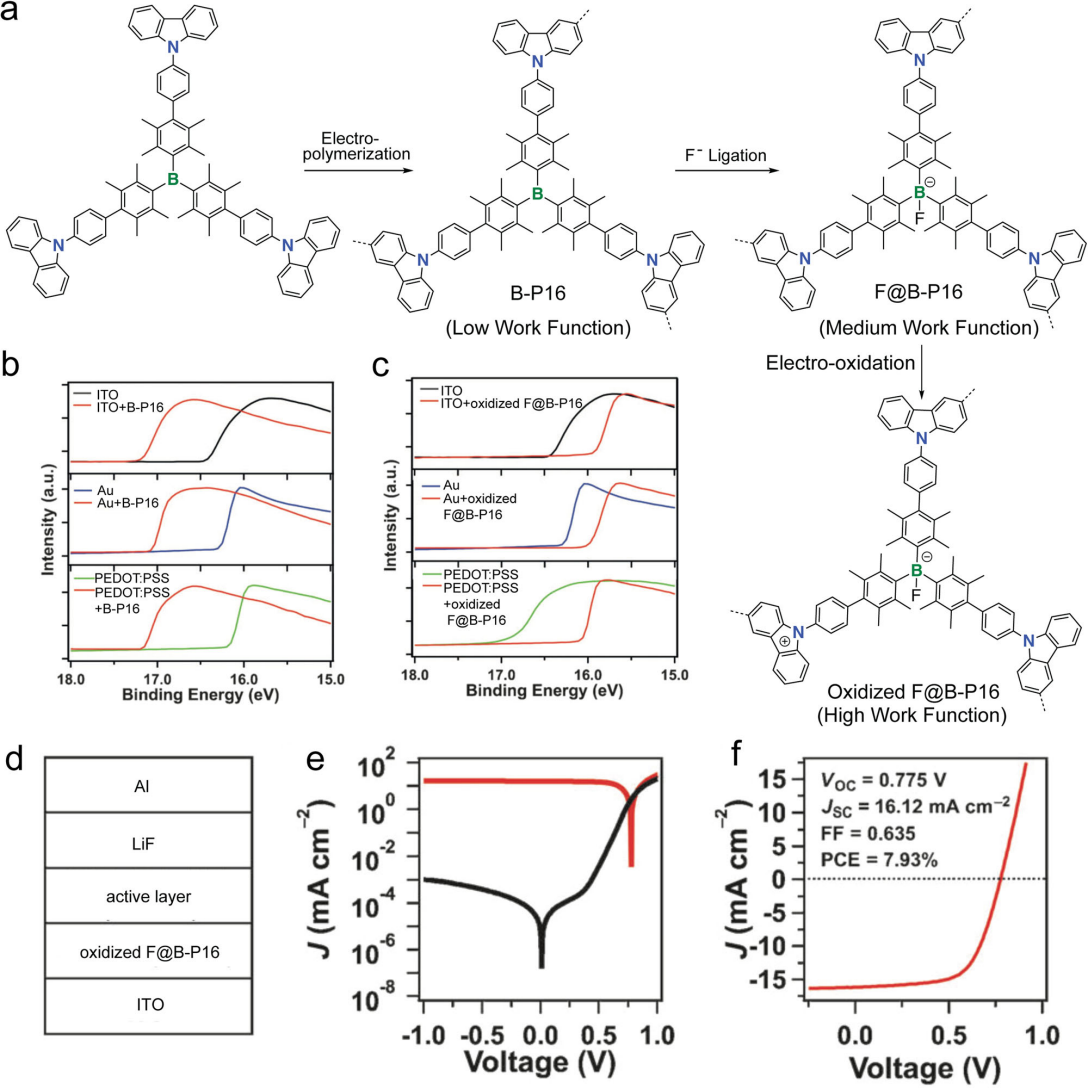
B-aPOPs synthesized by electro-polymerization. **a** In-situ electro-polymerization and the post-functionalization of B-P16. **b** Ultraviolet photoelectron spectroscopy spectra of various electrodes with and without B-P16 modification and (**c**) various electrodes with and without oxidized F@B-P16 modification. **d** Device structure of organic solar cells containing oxidized F@B-P16. **e** *J-V* characteristic in the dark (black) and under illumination (red). **f** *J-V* characteristics of the organic solar cell under AM 1.5 illumination (100 mWcm^−2^). (adapted with permission from ref. ^73^. © 2016 John Wiley and Sons).

In line with exploring new synthetic protocols, Jiang and coworkers successfully introduced the electro-polymerization protocol to construct B-*a*POPs on the surface of various electrodes (Fig. 6a)^73^. Based on the well-documented electro-polymerization of carbazole units in the literature, B-*a*POP (B-P16) with carbazole moiety was synthesized by harnessing the similar in-situ electro-polymerization. According to high-resolution transmission electron microscopy (HR-TEM) and Kr adsorption isotherm measurements, B-P16 exhibited the porous structure with a BET surface area of 1074 m^2^ g^−1^ and a pore size of 1.5 nm. Importantly, the surface polymerized B-P16 was able to effectively modify the work function of the various electrodes, such as ITO, Au, ZnO, and PEDOT:PSS. Leveraging on the Lewis acidity of the trivalent B-center, binding fluoride anion to B-P16 was further explored as a new strategy to elevate the work functions of the modified electrodes (Fig. 6b, c). Organic solar cells and light-emitting diodes using the modified ITO electrodes showed the excellent performances. Particularly, the organic solar cells based on oxidized F@B-P16 modified ITO exhibited a power conversion efficiency as high as 7.93% (Fig. 6d–f), which was the highest reported for the solar cells with porous polymers as interlayers. Overall, the research opened a new door for B-*a*POPs as new type materials to fine-tune the work function of the electrodes in various organic optoelectronic devices.

The developments of B-*a*POPs clearly showed two stages. In the first stage, the B-*a*POPs extensively borrowed the material design principles from the previous B-systems, including of using the protected B-building blocks, exploring the sensing properties of the B-*a*POPs towards fluoride and cyanide anions. Although some functionalized B-*a*POPs exhibited the promising catalytic properties in the oxidation of alkenes and homocoupling reactions of aryl Grignard reagents, the Lewis acidity character of the B-*a*POPs were rarely explored in new Lewis acid catalyzed synthesis. The second stage witnessed the advances of B-*a*POPs in the perspective of both new synthetic protocols and new applications. The B-Sn exchange reaction promised a new research direction that fully harness the Lewis acidity of the B-*a*POPs without the large protecting group. More applications of the B-*a*POPs in the Lewis acid catalyzed synthesis may become possible. With maturing of the structure design and synthesis, B-*a*POPs with new chemical structures were further tested in the various energy related applications, such as photocatalytic hydrogen production and electrode materials in organic electronic devices.

### Group 14 based amorphous POPs

**Fig. 7:**
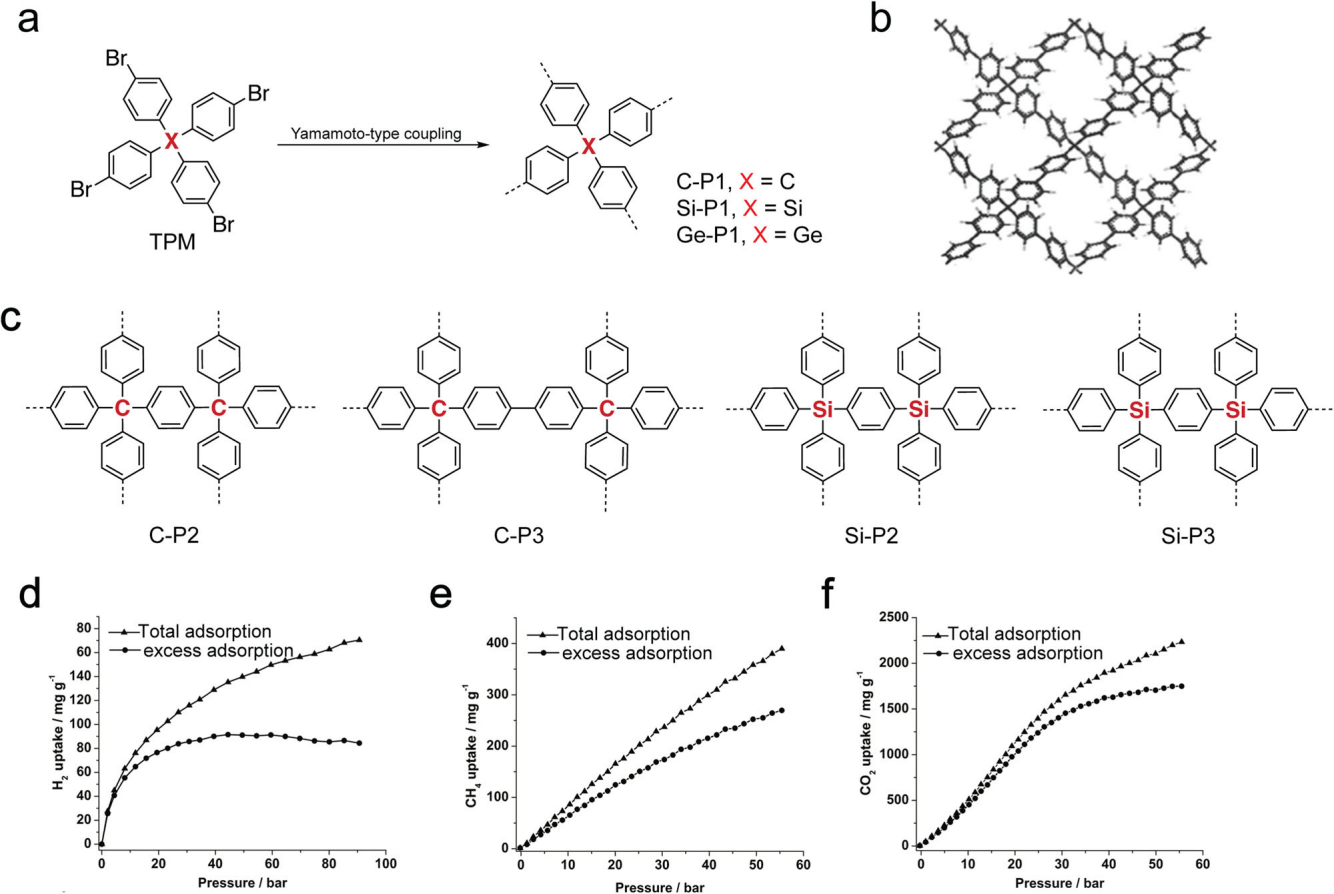
aPOPs with group-14 elements. **a** Synthesis of C-/Si-/Ge-*a*POPs containing various tetrahedral building blocks^10,60^. **b** Structure model of C-P1 (adapted with permission from ref. ^10^ © 2009 John Wiley and Sons). **c** Chemical structure of *a*POPs with various topological C-/Si-/Ge-building blocks in the literature. **d** H_2_ adsorption isotherms of Si-P1 at 77 K. **e** CH_4_ adsorption isotherms of Si-P1 at 295 K. **f** CO_2_ adsorption isotherms of Si-P1 at 295 K. (adapted with permission from ref. ^60^. © 2009 John Wiley and Sons).

In group 14, the light carbon element played very important roles in the early development of *a*POPs^1–3^. Not only the well-developed C–C coupling reactions provided the rich synthetic protocols, but also the introduction of tetrahedral C-center as the topological building block afforded C-*a*POPs with the stable porous structures and high surface areas. In 2009, Zhu and coworkers reported the first example of C-*a*POPs (C-P1 in Fig. 7a, b) containing tetrahedral TPM^10^. C-P1 was synthesized by the Nickel-catalyzed Yamamoto-type Ullmann coupling polymerization, which opened up a new subdiscipline of *a*POPs, namely PAFs. Due to the rigid 3D structure of TPM, C-P1 exhibits a BET surface area as high as 5600 m^2^ g^−1^. The excellent porosity and stability made C-P1 an promising candidate for gas storage applications, such as CO_2_ capture and H_2_ storage. C-P1 absorbed 1300 mg⋅g^-1^ CO_2_ at 40 bar at 298 K. The H_2_ uptake capacity of C-P1 (10.7 wt%, at 48 bar, 77 K) is comparable to the best performed MOFs and COPs at the time.

Following the research of C-P1, Zhou and coworkers reported several new examples of group-14 *a*POPs (Si-P1 and Ge-P1 in Fig. 7a) with tetrahedral Si-/Ge-building blocks^60^. As expected, Si-P1 and Ge-P1 exhibited high BET surface areas of 6461 m^2^ g^−1^ and 4267 m^2^ g^−1^, respectively. Particularly, Si-P1 showed the highest BET surface area among all porous materials reported at the time. Si-P1 also exhibited high H_2_ (91 mg g^−1^, 55 bar, 77 K, Fig. 7d), CH_4_ (389 mg g^−1^, 55 bar, 295 K, Fig. 7e), and CO_2_ (1710 mg g^−1^, 50 bar, 295 K, Fig. 7f) uptake capacity, which is very a promising candidate for the gas storage applications. Later, *a*POPs with various new topological C-/Si-/Ge-building blocks (Fig. 7c) were designed and synthesized, which also showed excellent porosity characteristics, such as high surface area and stable porous structure^3,33,74–77^. The results advocated the important role of the rigid 3D building blocks for designing *a*POPs with excellent porosity.

**Fig. 8:**
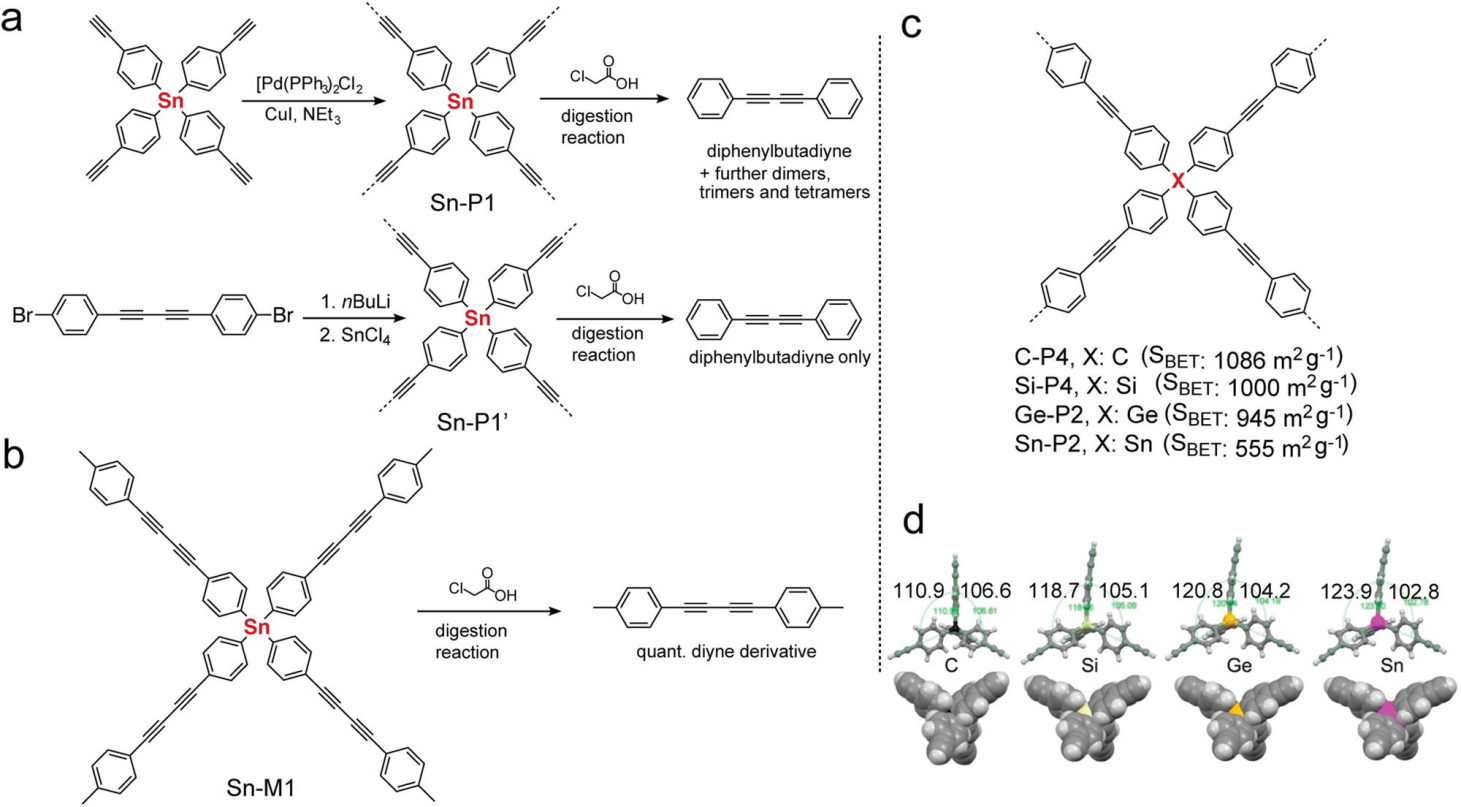
aPOPs with aryleneethynylene building block. **a** Synthesis of Sn-P1 and Sn-P1’ by using different synthetic protocols (adapted with permission from ref. ^78^ © 2018 John Wiley and Sons). **b** Digestion reaction of model compound Sn-M1. (adapted with permission from ref. ^78^. © 2018 John Wiley and Sons). **c** Chemical structure of C-P4, Si-P4, Ge-P2, and Sn-P2 in the literature^79^. **d** Crystal structures of the S-/Si-/Ge-/Sn-monomers for the synthesis of C-P4, Si-P4, Ge-P2, and Sn-P2 (adapted with permission from ref. ^79^ © 2018 John Wiley and Sons).

Recently, Bunz and coworkers reported a new type of Sn-*a*POPs (Fig. 8, Sn-P1) via Pd/Cu catalyzed homocoupling of tetrakis(4-ethynylphenyl)stannane^78^. Sn-P1 exhibits a BET surface area of 747 m^2^ g^−1^. As another important progress in *a*POPs, they were able to utilize liable Sn–C bond to probe the chemical compositions of Sn-P1. In the model digestion reaction (Fig. 8b), Sn-M1 was quantitatively converted to diyne derivative in the presence of chloroacetic acid. After applying a similar digestion reaction to Sn-P1’ synthesized by lithiation method (Fig. 8a), quantitative diphenylbutadiyne also was obtained. On the contrary, the digestion reaction of Sn-P1 only gave a small amount of diphenylbutadiyne (Fig. 8a). Most of the digestion products were the enyne-based dimers, trimers, and tetramers of phenyl-acetylene. The results suggested that the heterogeneous C–C cross-coupling polymerization did not always result in the uniform structures as we expected. This study provided a new strategy to elucidate the chemical structures of *a*POPs when they contain liable chemical bonds, such as Sn–C and Si–C bonds. Using similar Sn–C digestion method, Bunz’s group further investigated the chemical structures of similar Sn-*a*POPs synthesized under different reaction conditions. The acid-mediated digestion method further helped them uncover useful chemical structure information of the Sn-*a*POPs synthesized under different reaction conditions by changing catalysts, solvents, and bases^78^.

Later, the same group carried out the systematic studies on Group-14 *a*POPs (Fig. 8c, C-P4, Si-P4, Ge-P2, and Sn-P2)^79^. These *a*POPs exhibited a decreasing trend of BET surface areas with increasing atomic number of the central atom (C, Si, Ge, Sn). They hypothesized that the flexibility of the Group-14 building blocks has a strong impact on the BET surface areas, in which increasing atomic number and metallic character from C to Sn increased the flexibility. The observation that changing from C-center, over Si-center and Ge-center, to Sn-center showed the increasing deviation from the ideal tetrahedral angle is consistent with the hypothesis (Fig. 8d).

As shown in the section, the C-*a*POPs played an irreplaceable role and position in the developments of *a*POPs. The stable and rigid topological group-14 building blocks, particularly, C- and Si-building blocks, became the benchmark “legos” for constructing *a*POPs with high surface areas. The high stability of the C- and Si-building blocks also allowed the rich chemical modifications of the polymeric backbones, thus optimized the selectivity of the *a*POPs towards toxic chemicals, such as CO_2_ and ammonia^80,81^. Along with helping to maintain the excellent porosity, the group-14 building blocks further helped us to identify the chemical structures of *a*POPs. The study of the digestion reaction of the tetrakis(4-ethynylphenyl)stannane network clearly suggested that the chemical structures *a*POPs were not always uniform as we expected. Therefore, the chemical structures of new *a*POPs should be carefully studied, which is beneficial for establishing the clear and reliable relationship between chemical structure, properties, and functions.

### Group 15 Based Amorphous POPs

**Fig. 9:**
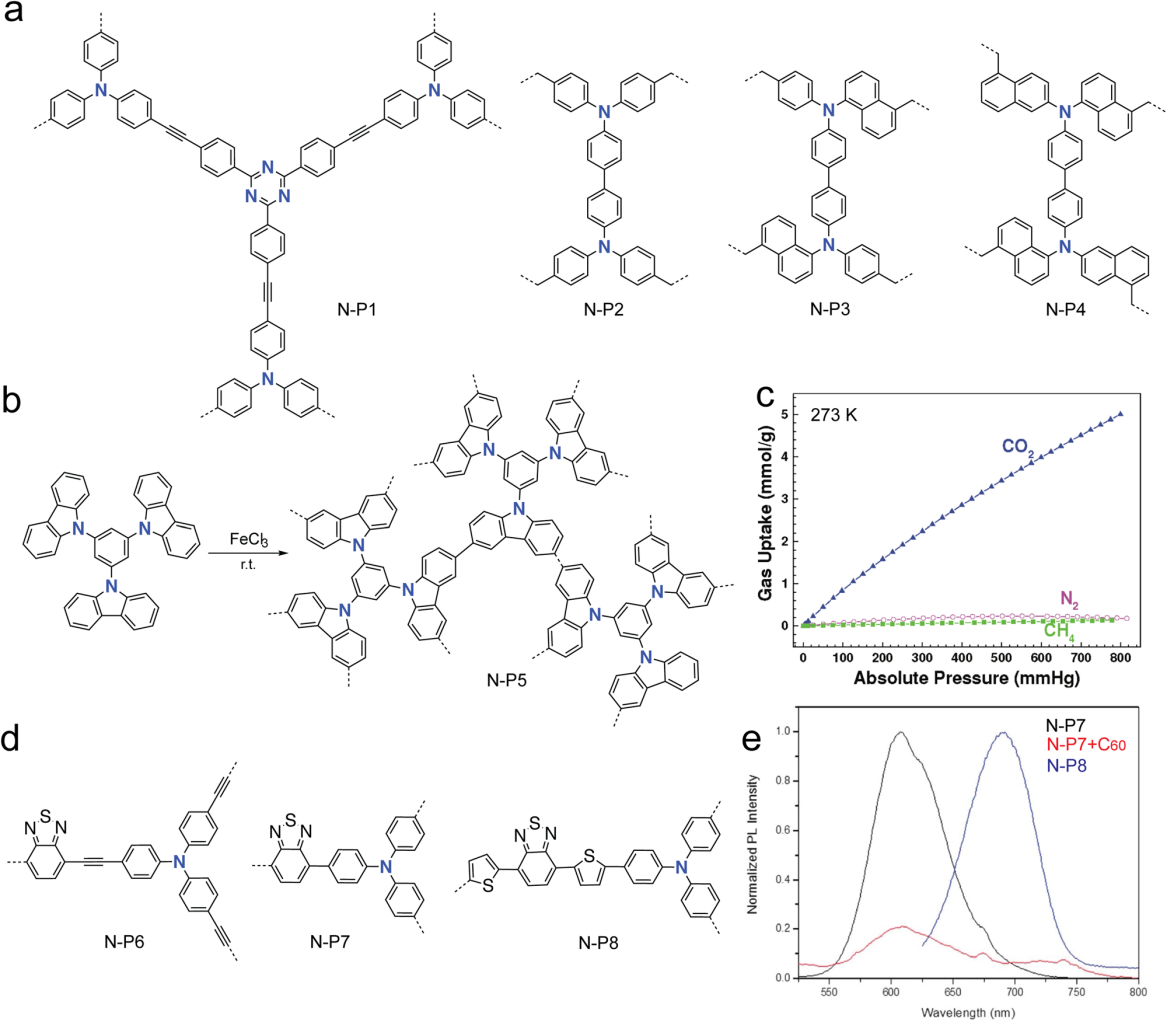
aPOPs with nitrogen element. **a** Chemical structure of N-P1-4^62,63^. **b** Synthesis of N-P5. **c** Gas adsorption isotherms of N-P5. (adapted with permission from ref. ^83^ © 2012 American Chemical Society). **d** Chemical structure of narrow band-gap N-P6-8. **e** Photoluminescence quenching spectra of N-P7 with C_60_ adapted with permission from ref. ^86^.

Similar to the above Group-14 derivatives, Group-15 element-based building blocks also exhibit nonplanar chemical structures. Incorporating Group-15 elements with lone pair electrons are expected to enhance the Lewis basicity of *a*POPs. Early reports revealed that N-*a*POPs (examples: N-P1-4, Fig. 9a) exhibited the promising Lewis acid gases (such as CO_2_, SO_2_, *etc*.) capture capability^62,63,82^. In a recent example, Han and coworkers applied oxidative coupling reaction to N-electron rich carbazole building block (Fig. 9b), which gave N-P5 with a BET surface area as high as 2220 m^2^ g^−1^ ^83^. N-P5 exhibited excellent hydrogen (2.8 wt %, 1.0 bar and 77 K) and CO_2_ (21.2 wt %, 1.0 bar and 273 K) storage capacities, which were competitive with the best reported results for porous polymers at the time. Furthermore, N-P1 showed a good selectivity toward CO_2_ over N_2_ and CH_4_, which also made them very promising materials for the gas separation (Fig. 9c). Later, more related N-*a*POPs were synthesized by using similar oxidative coupling polymerization, which showed promising photocatalysis performances in organic synthesis^84,85^. Band-gap engineerig of conjugated polymers is important for controlling the photophysical properties, redox properties, and energy conversions. Combining the electron-rich N-building block with electron-poor benzothiadiazole building block further resulted in new N-*a*POPs with narrow band-gap (N-P6-8, Fig. 9d)^86^. Compared with N-P6, N-P7 and N-P8 synthesized by the Suzuki coupling showed stronger photoluminescence. After doping with C_60_ as the electron-acceptor, the photoluminescence of N-P7 was significantly quenched (Fig. 9e). The studies suggested efficient charge transfer character in the N-P7-C60 complex, which could open up a new strategy for constructing efficient light harvesting or energy conversion architectures.

**Fig. 10:**
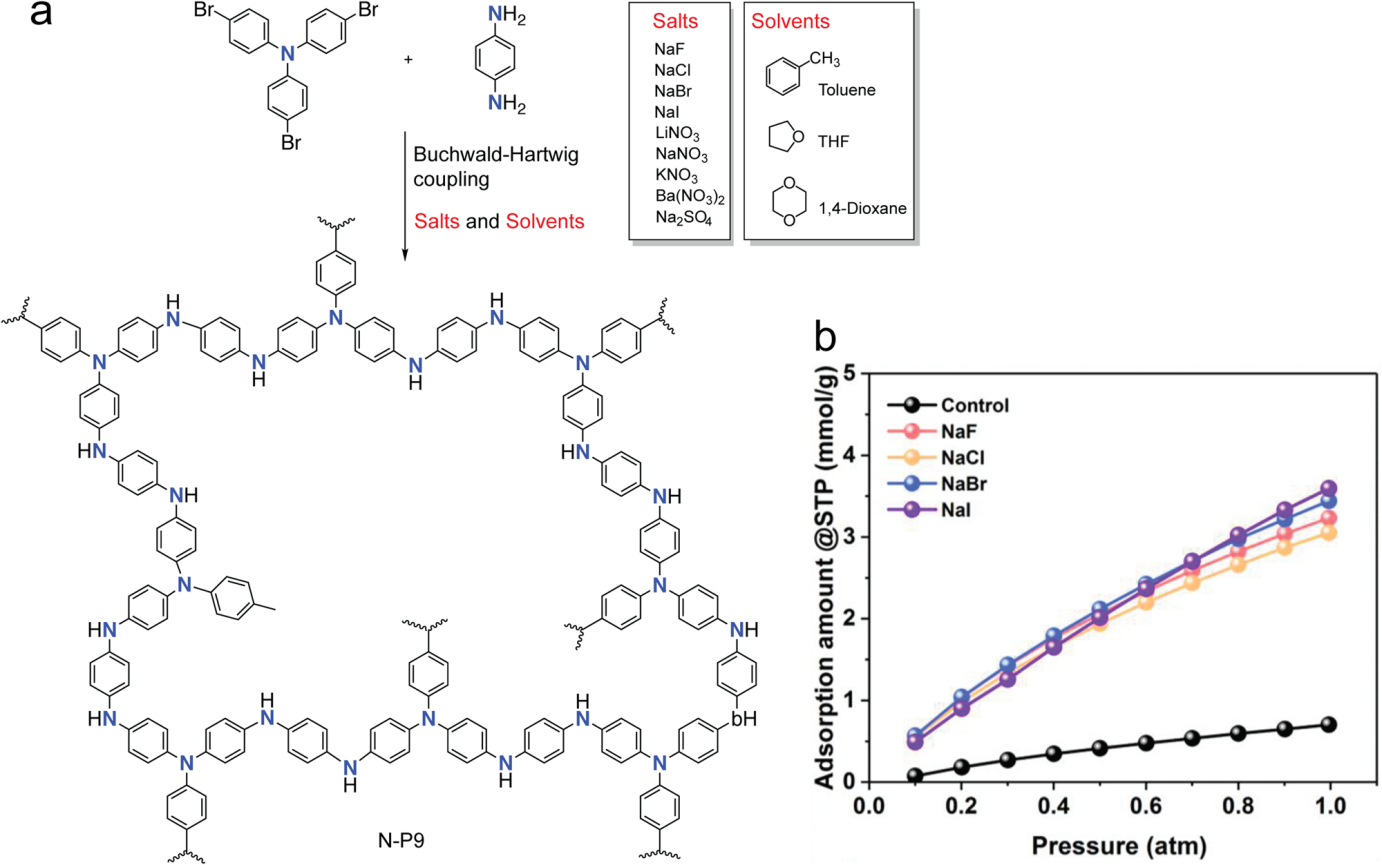
N-aPOPs synthesized by Buchwald-Hartwig coupling reaction. **a** Synthesis of N-P9 under various reaction conditions. **b** CO_2_ uptake at 273 K of N-P9 tuned by various salts^87^.

In a recent study, Faul and coworkers found that Buchwald-Hartwig (BH) cross-coupling reaction can be an efficient synthetic protocol for synthesizing N-*a*POPs (Fig. 10, N-P9)^87^. They found that the cross-coupling reaction conditions significantly influenced the porous structures of N-P9. With the presence of inorganic salts, such as sodium halides (NaF, NaCl, NaBr, and NaI), the BET surface areas of N-P9 increased to as high as 1152 m^2^ g^−1^ compared with that (58 m^2^ g^−1^) synthesized in the absence of salts. Along with the increased surface area, N-P9 synthesized in the presence of inorganic salts also exhibited a narrowed pore size distribution (PSD). The addition of the salts is believed to balance the Hansen solubility parameters (HSPs) between the polymers and the solvents, consequently results in the late-stage phase separation during the porous structure formation^88^. The improved porosity characteristics also enhanced the CO_2_ uptake capacity (from 0.70 mmol g^−1^ to 3.60 mmol g^−1^, 1.0 atm, 273 K, Fig. 10b).

**Fig. 11:**
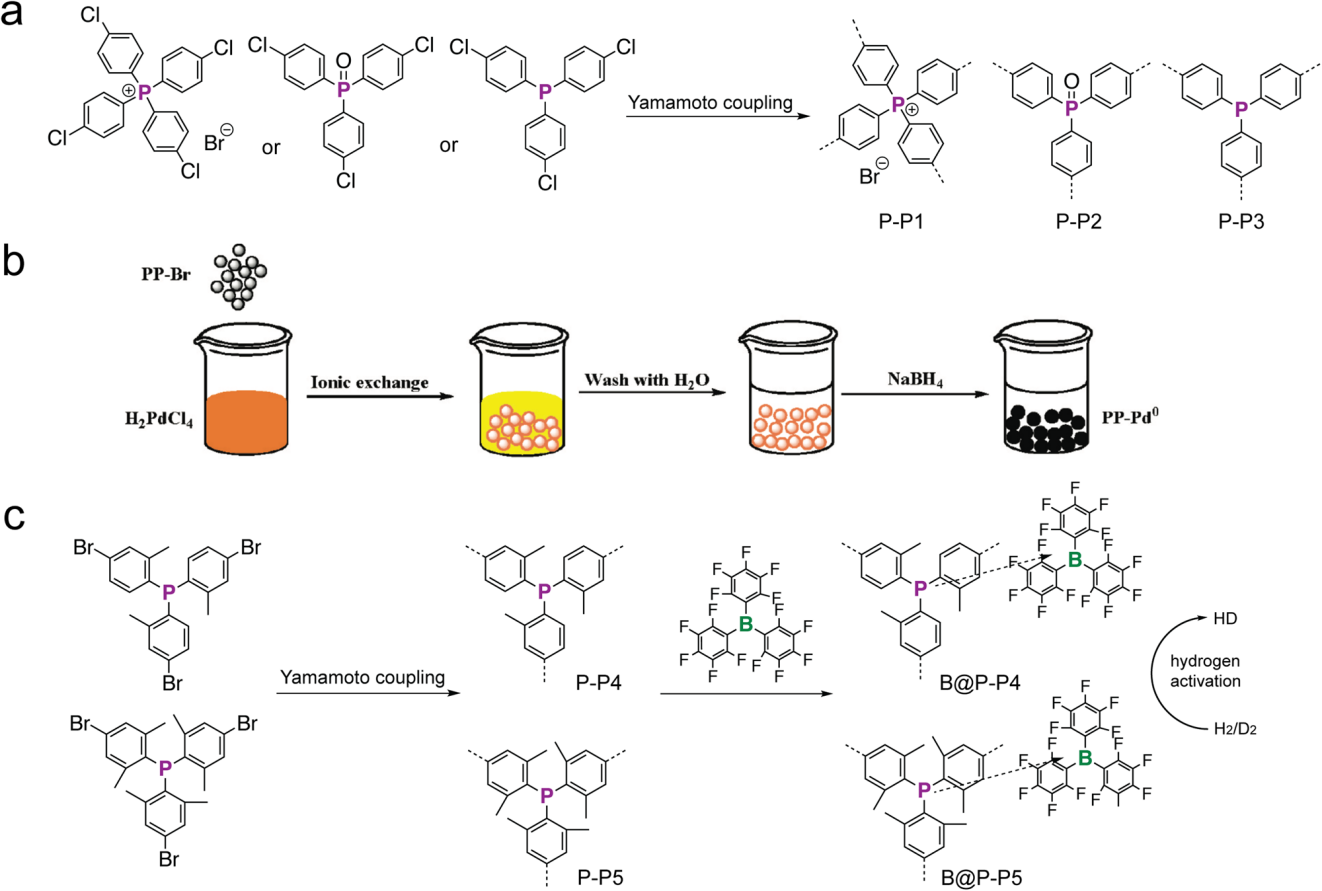
P-aPOPs with various P-environments. **a** Synthesis of P-P1, P-P2, and P-P3^91,92^. **b** Depositing Pd nano-particle via the ionic exchange reaction. (adapted with permission from ref. ^91^ © 2016 American Chemical Society). **c** Synthesis of P-P4, P-P5 and hydrogen action of B@P-P4, and B@P-P5.98.

In Group 15, rich phosphorus chemistry, such as oxidation, borylation, alkylation, metal coordination, *etc*., allowed to further enrich the chemical structures and properties of small molecules and macromolecules^89,90^. Using classical C–C cross-coupling polymerizations, diverse building blocks with various P-centers, such as P-lp (lp: lone pair of electrons), P-O, P-S, cationic P-R centers, P-metal centers were designed to synthesize P-*a*POPs in the literature^91–93^. Zhang and coworkers reported the first example of P-*a*POPs with a quaternary phosphonium center (Fig. 11a)^91^. Similar to TPM-type building blocks, the tetrahedral phosphonium cation building block was polymerized via the Yamamoto-type cross-coupling protocol, thus affording the target P-P1. Solid state ^31^P NMR spectra of P-P1 showed two signals at 23 ppm and −8.8 ppm. The former peak was assigned to the quaternary phosphonium center. While, the later high-field peak was attributed to the tertiary P-lp center, which was rationalized to be due to the P–C bond cleavage as the side reaction in the polymerization. The BET surface areas of P-P1 were highly tunable via the ionic exchange of counter anions. Decreasing the counter anion size from Br (650 m^2^ g^−1^) to Cl (750 m^2^ g^−1^) and F (980 m^2^ g^−1^) increased the BET surface areas of the P-*a*POPs. After depositing Pd nano-particle via the ionic exchange reaction (Fig. 11b), Pd@P-P1 exhibited a good catalytic C–C cross-coupling performance in the reaction between aryl halides and phenylboronic acid. Furthermore, similar catalytic reaction was even applicable to phenyl fluoride derivative by using Pd@P-P1 as the catalyst. Later, the same group also synthesized P-P2 with P-lp center and P-P3 with P-O center by using similar Yamamoto-type reactions^92^. Both P-P2 and P-P3 showed high BET surface areas. Pd@P-P2 with deposited Pd nanoparticle showed good catalytic performance in the coupling reactions between aryl chlorides and *p*-tolylboronic acid.

Although a great number of Lewis basic and Lewis acidic porous polymers were reported in the literature, these porous polymers were rarely applied for catalytic applications. In 2017, Thomas and coworkers reported two new semi-immobilized frustrated Lewis pairs *a*POPs (Fig. 11c, B@P-P4 and B@P-P5) and explore their potential catalytic applications^94^. B@P-P4 and B@P-P5 were prepared by mixing Lewis basic P-*a*POPs (P-P4 and P-P5) and Lewis acidic tri(pentafluororophenyl)borane (B(C_6_F_5_)_3_) additive. Interestingly, solid state ^31^P NMR spectra of B@P-P4 and B@P-P5 show down-field shifted signal compared to these of P-P4 and P-P5. Generally, the formations of frustrated Lewis base-acid adducts are not observed at ambient temperature in solution. It was rationalized that the favorable interactions gained from the weak association of the polymer and B(C_6_F_5_)_3_ dominated by the entropic contribution of the dissociation process. Importantly, B@P-P4 and B@P-P5 are able to cleave dihydrogen heterolytically at ambient temperature and low hydrogen pressure (Fig. 11c).

**Fig. 12:**
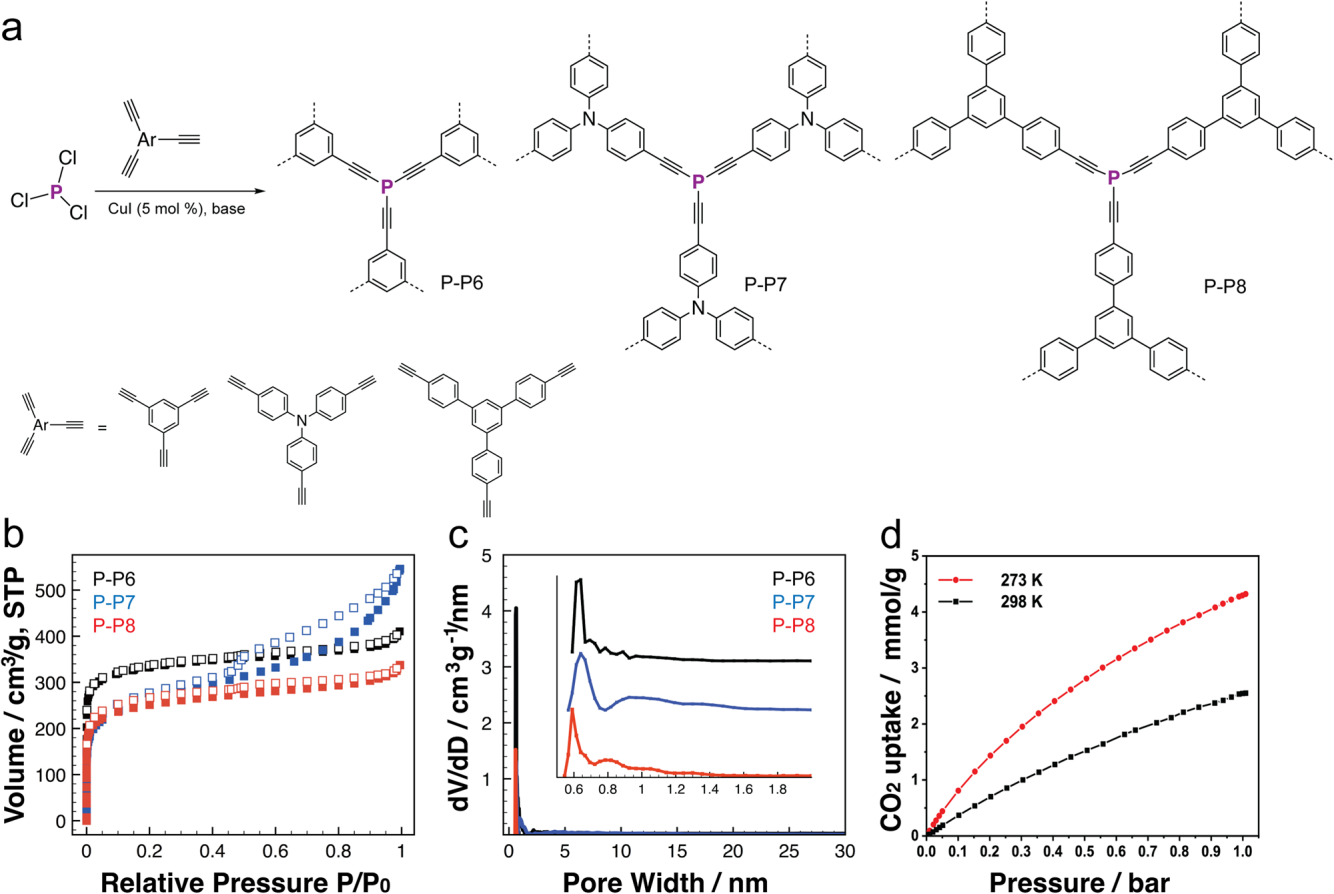
P-aPOPs synthesized by CuI catalyzed P–C coupling reaction. **a** Synthesis of P-P6, P-P7, and P-P8. **b** N_2_ sorption isotherms measured at 77 K. **c** Pore size distribution curves. **d** CO_2_ uptake of P-P6 at 273k and 298 K. (adapted with permission from ref. ^58^ © 2012 American Chemical Society).

Very recently, Ren’s group reported a new series of P-*a*POPs containing Phospha-alkyene structure (Fig. 12a, P-P6, P-P7, and P-P8)^58^. Unlike previous *a*POPs synthesized by C–C cross-coupling reactions, P-P5, P-P6, and P-P7 were synthesized by CuI catalyzed P–C cross-coupling reaction between PCl_3_ and various aryl alkynes. P-*a*POPs exhibited high surface areas (ca. 1000 m^2^ g^−1^) and ultra-microporous structure with PSD as small as 6.7 Å (Fig. 12b, c). More importantly, the study revealed that the choice of organic bases has the strong impact on the degree of crosslinking P-centers, thus resulting in the different porosities. CO_2_ uptake of P-P6 with most uniform P-crosslinking centers and highest microporous surface are is as high as 4.23 mmol g^−1^ (1 bar, 273 K, Fig. 12d), which is highest value among the polymers. The study provides important information on the relationship between the crosslinking environments, porous structures, and CO_2_ uptake capability, in which the higher the percentage of crosslinked P-centers, the higher the micro-surface areas of P-*a*POPs, and higher the CO_2_ uptake capability.

**Fig. 13:**
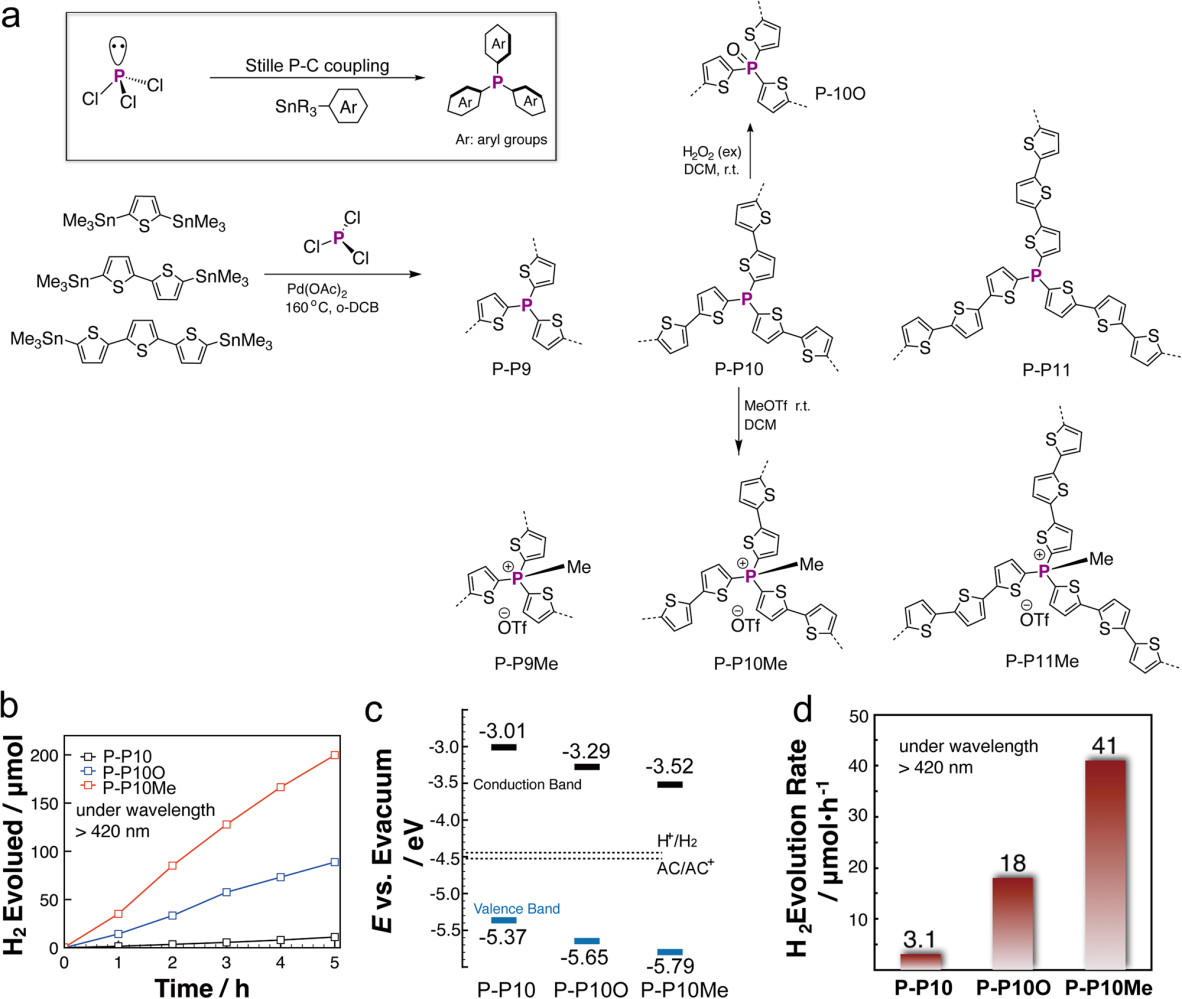
P-aPOPs synthesized by Stille type P–C coupling reaction. **a** Synthesis of P-P9, P-P10, and P-P11. **b** Time course for photocatalytic H2 evolution using 20 mg of photocatalysts. **c** Band structure diagram of the P-P10 series. **d** Photocatalytic H_2_ evolution rates. (adapted with permission from ref. ^95^ © 2023 Royal Society of Chemistry).

Following the CuI catalyzed formation of P-*a*POPs, Ren’s group recently extended the classical Stille C–C coupling reaction to the P–C bond formation where three P–C bonds are able to form at a single P-center (Fig. 13a)^95^. Upon the oxidation of P–C bond, electrophilic character of catalytic Pd(II) center was suppressed by the electron-rich P-center, which is distinct from the classical Still coupling reaction. The efficient P–C bond formation allowed us to synthesize a new series of P-*a*POP (P-P9, P-P10, and P-P11, Fig. 13b). Various P-chemistry was also applicable to the systems that gave the polymers with phosphonium center (P-P9Me, P-P10Me, and P-P11Me, Fig. 13b), and the polymer with P(O)-center (P-P10O, Fig. 13b). Although not showing microporours structures, the P-*a*POPs exhibited the promising visual-light photocatalytic hydrogen production properties (Fig. 13b). Compared with those of P-P10 and P-P10O (Fig. 13c), the lower valence band energy of P-P10Me is benificial for hole transport from sacrificial electron donor to the polymer. More importantly, adjusting the chemistry P-center of the polymers offered a new strategy to fine-tune the photocatalytic H_2_ production properties (Fig. 13d). P-P10Me with an ionic P(Me)-center exhibit a H_2_ evolution rate up to 2050 mmol h^−1^ g^−1^, which is much higher than those of P-P10 with P(O)-center (900 mmol h^−1^ g^−1^) and P-P10 with P(III)-center (155 mmol h^−1^ g^−1^).

The previous studies clearly showed that the lone pair of electrons and non-planar structures of group-15 elements furnished new functions and opportunities to *a*POPs. For example, introducing N-element into B*-a*POPs endowed the excellent responsive photoluminescence towards fluoride and cyanide anions^96^. The presence of N- and P-elements with strong Lewis base characters in the backbones improved the CO_2_ capture and H_2_ splitting of the GM-POPs^58,97^. Furthermore, the group-15 *a*POPs also found new platform in the applications of photocatalysis^85^. The study that the tunable visible light photocatalytic H_2_ production by the rich P-chemistry of P-P10 series clearly promise more opportunities for the photocatalytic applications^95^. The very recent studies of the group-15 *a*POPs also shed light on how the reaction conditions (such as solvents, temperature, catalysts, bases, etc.) affects the crosslinking centers, porous structures, and properties of the *a*POPs^58,87^.

### Group 16 element based amorphous POPs

**Fig. 14:**
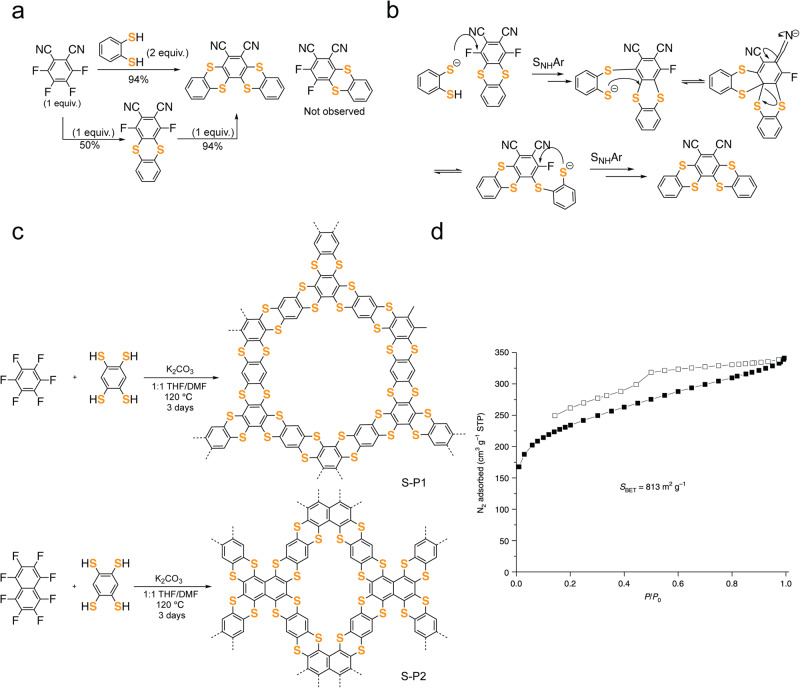
S-aPOPs synthesized by dynamic SNAr reaction. **a** Model reaction illustrating the dynamic, self-correcting nature of the S_N_Ar reaction. **b** Proposed mechanism for the reaction. **c** Synthesis of S-P1 and S-P2. **d** N_2_ sorption isotherms of S-P2 measured at 77 K. (adapted with permission from ref. ^61^ © 2023 Royal Society of Chemistry).

Lately, group-16 element chemistry also brought some intriguing aspects into the filed of *a*POPs in the perspectives of exploring new synthetic protocols and functions. In 2018, Swager and coworkers reported the use of nucleophilic aromatic substitution reaction (S_N_Ar) to construct S-*a*POP^61^. They found that the dynamic and self-correcting nature of the S_N_Ar between ortho-aryldithiols and ortho-aryldifluorides is responsible for the regioselectivity of thermodynamic products (Fig. 14a, b). Applying the S_N_Ar in polycondensation, they were able to obtain new S-*a*POPs (Fig. 14c) with BET surface areas of 189 m^2^ g^−1^ for S-P1 and 813 m^2^ g^−1^ for S-P2 (Fig. 14d), respectively. Recent studies also showed that similar dynamic S_N_Ar polycondensations between phenol derivatives and activated aryl halides gave O-based COFs with highly ordered structures by the careful optimization of polymerization conditions^98^. The detailed discussion of these studies is out of the scope of this review.

**Fig. 15:**
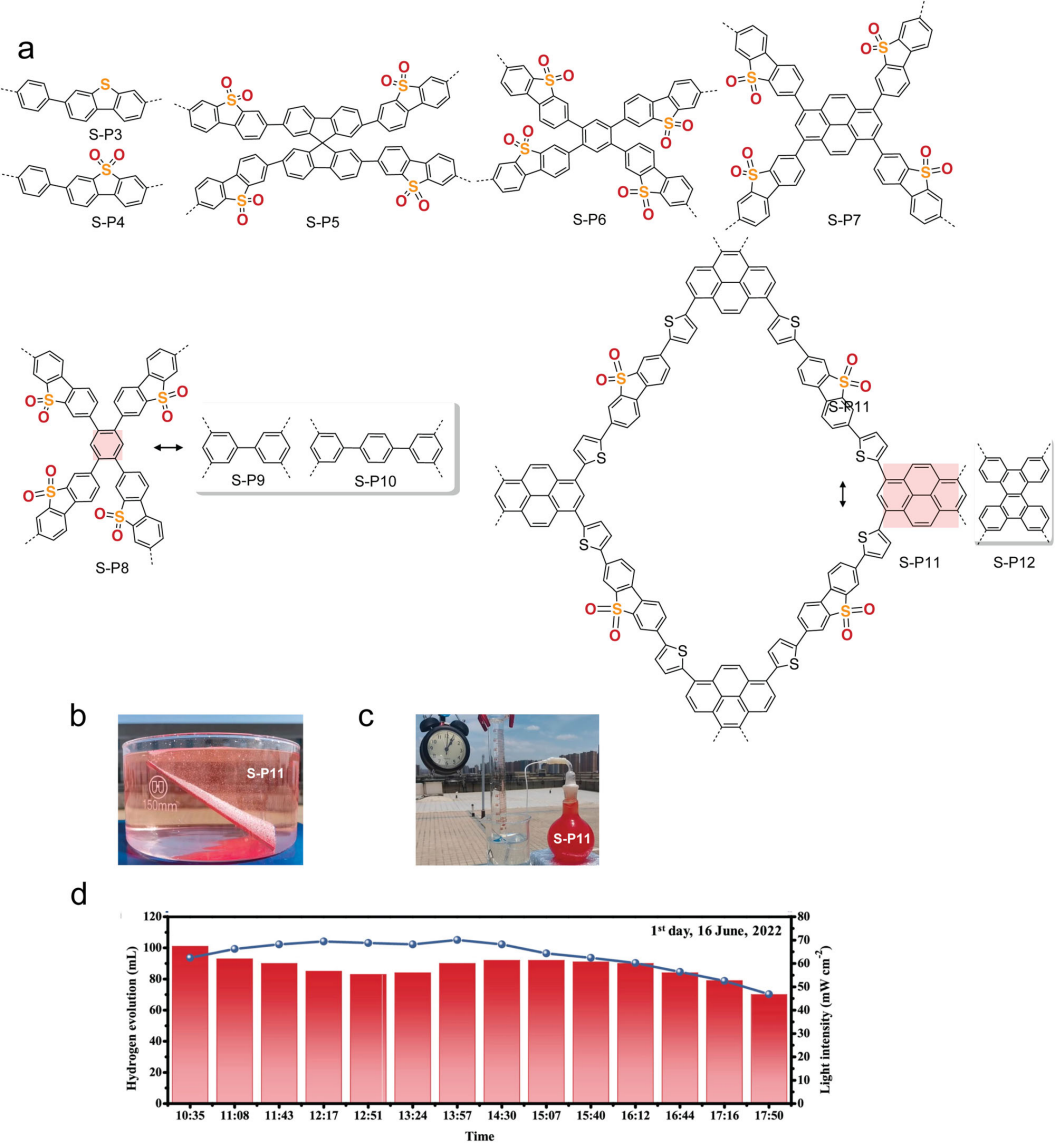
aPOPs with sulfone group. **a** Structures of S**-***a*POPs showing photocatalytic hydrogen production properties in the selected literature. **b** S-P11 film for hydrogen generation under natural light. (adapted with permission from ref. ^104^ © 2012 American Chemical Society). **c** Home-made equipment for water-drainage and gas-gathering experiments under natural light. (adapted with permission from ref. ^104^ © 2012 American Chemical Society) **d** Time course of hydrogen evolution for S-P11 with 3 wt% Pt under natural sunlight. (adapted with permission from ref. ^104^ © 2012 American Chemical Society).

In addition to showing the intriguing synthetic reactivities, the introduction of S-center was also found to endow S-*a*POPs with photocatalytic H_2_ production properties. In 2016, Cooper and coworkers found that converting S-center to sulfone (SO_2_) center in linear conjugated polymers (Figs. 15a, S-P3,4) significantly enhanced the photo-hydrogen evolution performance, in which HER up to 5.8 mmol h^−1^ g^−1^ was obtained for the SO_2_-containing conjugated polymer^99^. Encouraged by the promising photocatalytic H_2_ production properties, Cooper and coworkers further synthesized several series of S-*a*POPs (Examples shown in Figs. 15, S-P5,6) where SO_2_-*a*POPs consistently exhibited better photocatalytic hydrogen production performance compared with their S-*a*POP counterparts^100^. The ultrafast spectroscopy and theoretical studies suggested the several important roles of the sulfone group played in the photocatalytic properties:^101,102^ (a) The presence of sulfone groups improved the hydrophilic character of polymeric backbones, in which the charge and proton transfer could be accelerated at the interface between the polymeric backbones and water/triethylamine molecules. (b) The polymer containing SO_2_-group exhibits the improved charge transfer and triethylamine deprotonation, presumably due to the high polarity of sulfone group. (c) The yields of polaron generated by photoexcitation increase with the number of SO_2_-groups in the polymeric backbones. Recently, Jiang and coworkers reported new series of S-*a*POPs (Figs. 15, S–P7–12)^103–105^. By judiciously choosing organic π-conjugated building blocks, they were able to synthesize SO_2_-*a*POP (S-P12) with HER as high as 115 mmol h^−1^ g^-1^ under visible light (*λ* > 420 nm)^104^. Jiang’s group further demonstrated that, under natural sunlight, the polymer-film based S-P12 were also highly active in the outdoor photocatalytic H_2_ production (Fig. 15b, c, d). The total 1224 mL of H_2_ and an everage HER of 312 mmol h^−1^ g^−1^ was achieved under the irradiation of natural sunlight for 7 h (Fig. 15b–d)^104^.

As shown in the section, the group-16 elements were not generally used for constructing the *a*POPs with good porous stuctures (such as high surface areas and uniform pore sizes) compared with the other MG-*a*POPs. Altough the dynamic chemistry between ortho-aryldithiols and ortho-aryldifluorides brought some interesting porosity for *a*POPs, the universality of the chemistry limited the developments of new MG-*a*POPs with the excellent porous stuctures. At contrary, the group-16 *a*POPs were extensively investigated in the application of photocatalytic H_2_ production, which hold promise for advancing the photocatalytic applications.

### Exploring new synthetic protocols for MG-Amorphous POPs

Rich organic synthetic methods provided a large pool of synthetic protocols for constructing *a*POPs with various chemical structures, porous structures, and properties. The “mix and polymerize” strategy powered by the rich synthetic chemistry are very appealing, which attracted both chemistry and materials researchers into the field of *a*POPs, including MG-*a*POPs. For example, the synthetic methods based on transistion metal catalyzed C–C cross-coupling reactions were benchmarked for introducing diverse functional building blocks into MG-*a*POPs.

To expand the diversity of chemical structures and properties, recent research of MG-*a*POPs has been mainly focusing on the design of new building blocks with more complex structures. Although the diverse building blocks enriched the structures and functions, these complex building blocks generally required multiple-step syntheses and non-trivial purification processes. Therefore, it is envisioned that using simple and commercially available MG-building blocks could be alternative strategy for considerably streamlining the synthesis and diversifying the properties of *a*POPs. For example, Ren’s group was able to use BBr_3_ and PCl_3_ as the simple starting materials to build MG-*a*POPs with active MG-centers^39,58^. It was found that the B–Sn exchange reaction and CuI catalyzed P–C coupling reaction are highly efficient at the single B-atom and P-atom, which afforded new MG*-a*POPs with good porous characteristics.

It is worthy of mentioning that Kaskel’s group and Hausoul group used similar halides (PCl_3_ and SiCl_4_) and aryl-lithium and aryl-Grignard reagents as the starting materials to construct MG*-a*POPs^106–108^. However, the use of highly reactive organometallic reagents limited the scope of starting materials that are sensitive to the strong nucleophilic organometallic reagents. In another recent example, Chan and coworkers reported a new series of Bi-containing crosslinked organic polymers by using Bi–S polycondensation^109,110^. Although the porosimetry measurements revealed the low surface areas of the Bi-polymers, the study is a nice demonstration of the heavier main-group chemistry in the synthesis of MG-*a*POPs. However, the high toxicity of heavier main group element Bi should also be paid attention.

**Fig. 16:**
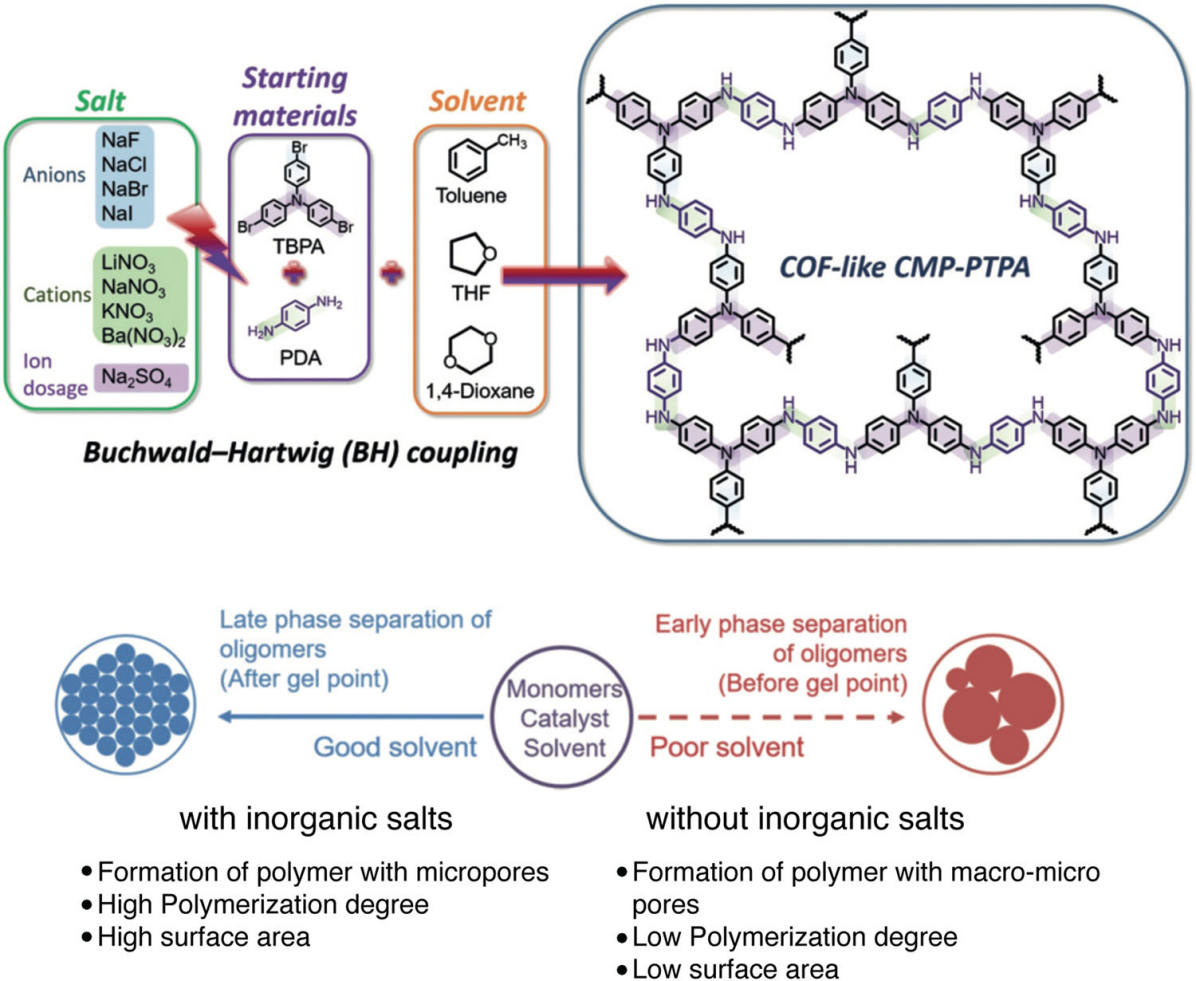
Proposed inorganic salt effects in the polymerization of N-P8. (adapted with permission from ref. ^87^ © 2019 John Wiley and Sons).

Different from the synthesis of *c*POPs generally involved reversible bond-forming and bond-breaking processes,(MG*-*)*a*POPs are synthesized by irreversible chemical bond formation^1–3,111–116^. The intrinsic bond formation mechanism of (MG*-*)*a*POPs is believed to be detrimental to constructing the well-controlled chemical and porous structures. The nature of heterogeneous polymerization condtions further restricted the better controls in the chemical and porous structures. Recent studies that identified some important reaction characteristics for the better-controlled chemical and porous structures of *a*POPs are highly appealing. For example, Faul’s group discovered that the inorganic salts have the strong impacts on the BET surface areas and PSD of N-P8 synthesized by Buchwald-Hartwig reaction (Fig. 16)^87^. They proposed that the salts act similarly to the addition of porogens, which helps to tune the HSPs between polymers and reaction solvents. According to the classic HSP principle, solvent with poor thermodynamic compatibility and weak matching of the HSPs with the resultant polymeric networks could lead to the formation of microgels and early phase separation. In this situation, the *a*POPs with large average diameter pores and low surface areas were rationalized to be obtained. The addition of the salts is believed to match the HSPs between the polymers and the solvents, consequently resulting in the late-stage phase separation of the polymerization. Therefore, N-P8 with lower PSD and larger surface area was expected to be obtained in the presence of the salts. The hypothesis was further supported by the calculation of the HSP in various solvents. This mechanism also explains that the permanent dipole interactions and the hydrogen-bonding interactions of the ions enhance with the decrease of their ionic radius, consequently giving a better adjustment of the compatibility of the HSPs with the polymers.

**Fig. 17:**
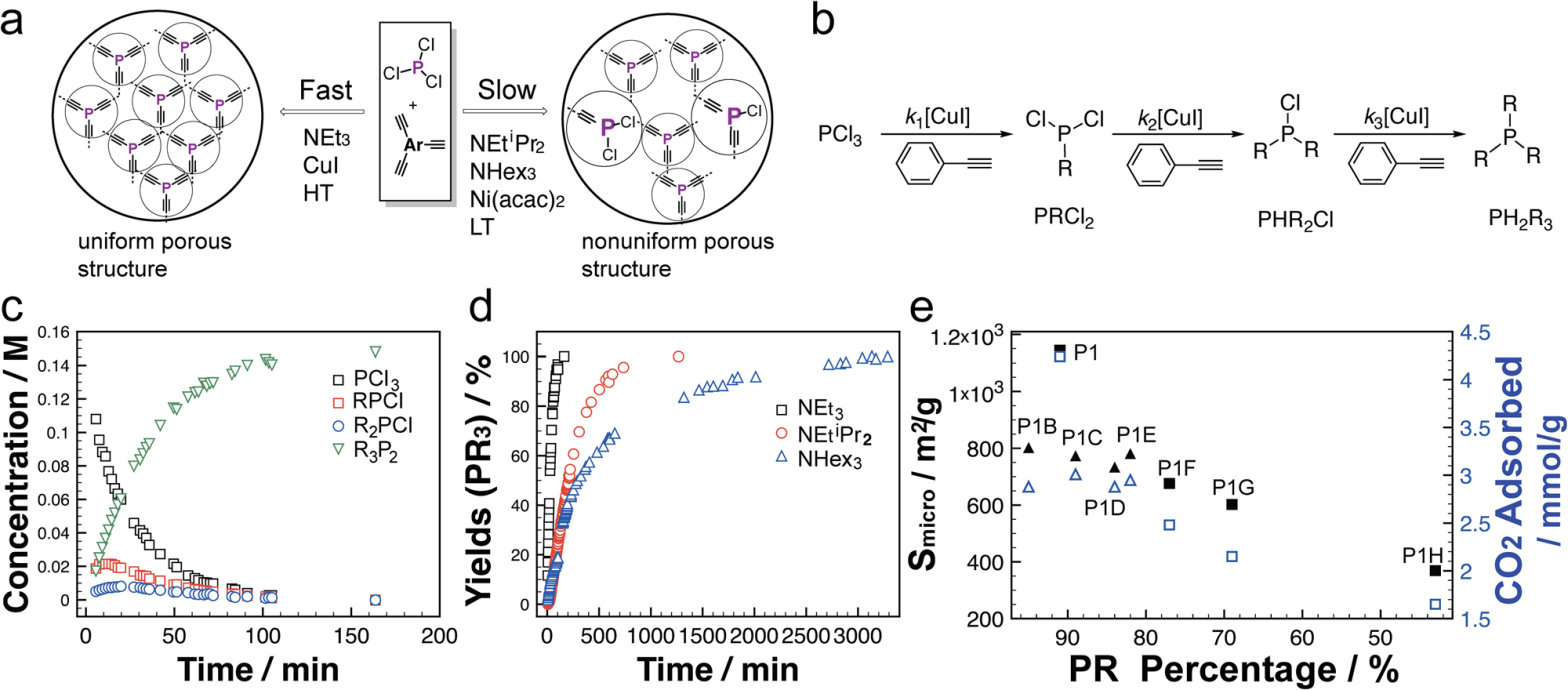
Kinetic effects in the P–C coupling reaction. **a** In fluence of P − C coupling kinetics under various reaction conditions on the chemical structures and porosity. **b** Stepwise illustration of the model reaction. **c**, **d** Kinetic NMR studies of the P−C coupling in the presence of various bases. **e** The correlations between the crosslinked P-centers, S_micro_ and CO_2_ uptake capacity. (adapted with permission from ref. ^58^. © 2022 American Chemical Society).

In another recent example, Ren’s group identified that the self-accelerating kinetic character of Cu-catalyzed P–C coupling reaction is beneficial for constructing the better-controlled and better-defined crosslinking environments and porosity in P-*a*POPs (Fig. 17a)^58^. In the kinetic study of model reactions, the reaction rate constant *k*_3_ is higher than the reaction rate constants *k*_1_ and *k*_2_ (Fig. 17b, c). Furthermore, the choice of base also influnenced the kinectic of the model reactions (Fig. 17d). In line with the model studies, the P−C polymerizations with the high reaction-rate conditions afforded P-*a*POPs with more uniform crosslinking environments, better-controlled porosity, high ultramicroporous surface areas, and high CO_2_ uptake (Fig. 17e). More fully crosslinked P-centers are expected to be achieved for a reaction with the ideal self-accelerating characteristic (*k*_3_ ≫ *k*_1_, *k*_2_) where three P–C bonds at a single P-center almost instantly form at the same time. In the ideal scenario, the local crosslinked microporous environments may form uniformly within a very short time scale. It is believed that other types of reactions with the similar kinetic reaction characteristics may be also applicable to the synthesis of *a*POPs with better-controlled chemical and porous structures.

### Exploring experimental characterization techniques for MG-Amorphous POPs

Experimental characterization techniques are very useful to shed light on the relationship between chemical structures, porosity and functions of MG-*a*POPs, which will accelerate the finding of new strategies for designing new functional MG-*a*POPs. All the experimental characterization techniques used in *a*POPs are also applicable to MG-*a*POPs. Like typical porous materials, gas sorption experiments are the main characterization technique to provide the porous structure information, such as surface area, porous diameter, and shape of porous structures in MG-*a*POPs^117–121^.

The chemical composition of MG-*a*POPs can be readily examed by the well-established materials characterization techniques, such as Fourier transform infrared (FTIR), elemental analysis (EA), inductively coupled plasma optical emission spectroscopy (ICP-OES), X-ray photoelectron spectroscopy (XPS), and solid state NMR. In addition to revealing the chemical compositions, these techniques with appropriate modifications were also able to uncover useful information on the porous structure formation of MG-*a*POPs. For instance, in-situ FTIR experiments were used to track the process of C–C bond forming in the porosity formation progress of C-*a*POPs^111^. In another example of the “digesting and probing” method, ^1^H NMR experiments of the digested Sn-*a*POPs indirectly identified the chemical compositions^78,122^. The results of these modified experiments clearly suggest that the polymerizations of the *a*POPs were not always as uniform as expected, and various side reactions may occur during the polymerizations of monomers.

**Fig. 18:**
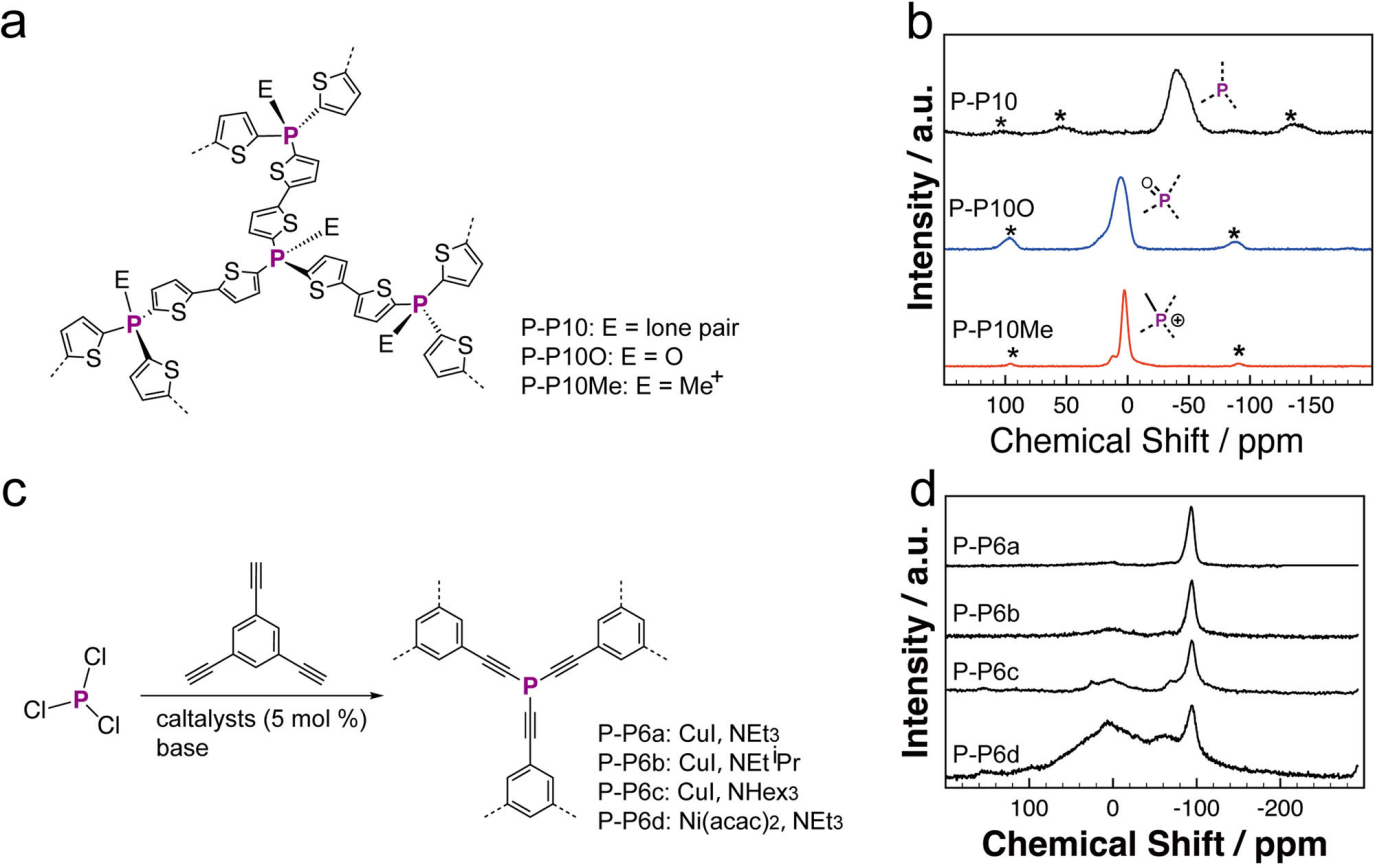
Solid state 31P NMR studies for P-aPOPs containing various P-environments. **a** Chemical structure of P-P10 series with various P-center. **b** Solid state ^31^P NMR spectra of P-P10 series. (adapted with permission from ref. ^95^ © 2023 Royal Society of Chemistry). **c** Synthesis of P-P6 series under different conditions. **d** Solid state ^31^P sNMR spectra of P-P6 series under different conditions. (adapted with permission from ref. ^58^ © 2012 American Chemical Society).

Solid state multinuclear (^11^B, ^31^P, ^19^F, etc.) NMR spectroscopy is another powerful technique that is able to provide the important chemical information of *a*POPs containing NMR active MG elements, such as the chemical purifty and valence of the MG-centers. For example, solid state ^11^B and ^31^P NMR experiments were widely used to identify the presence of various B centers and P centers in MG-*a*POPs^39,58,95^. Compared with those of solid state ^13^C NMR experiments, the signals of ^11^B and ^31^P NMR experiments are more sensitive to the chemical environments. The ^11^B and ^31^P NMR experiments were also able to track the chemical environment changes of the element centers^39,58,95^, such as Lewis acid-base interactions at the B-center (Fig. 4d) and the chemical valence of P-centers (Fig. 18a, b).

Although the chemical compositions of MG-*a*POPs, like all the *a*POPs, can be well characterized by the typical material characterization techniques mentioned above, precisely probing the solid-state structures of MG-*a*POP are still very challenging. With the intrinsically amorphous nature, the solid-state structures of *a*POPs including MG-*a*POPs cannot be investigated by X-ray diffraction experiments. Without the reliable structural information, the relationship between structures, properties and functions of MG-*a*POPs are hard to be generalized, which further limited the design of new MG-*a*POPs with improved properties.

In the solid-state structure, the environments of crosslinking centers not only play important roles in determining the chemical uniformity, but also governing the porous structures of *a*POPs including MG-*a*POPs. Very recently, characterizing the chemical environments of crosslinking centers in MG-*a*POPs became possible by using various NMR active MG elements (such as B and P) as the direct crosslinking centers in the synthesis, which readily shed light on the solid-state structures. Although solid state ^13^C NMR experiments were successfully used to qualitatively track the polymerization progress of C-*a*POPs in a previous study^115^, the characteristics of weak solid state ^13^C NMR signals and possible indistinguishable peaks of the probed C-centers (mostly sp^2^ and sp^3^ carbons) with other similar C-centers make the solid state ^13^C NMR experiments rarely provided quantitative information on the chemical environments of the crosslinking C-centers. Leveraging on the highly sensitive solid state ^31^P NMR signals under various environments, solid state ^31^P NMR experiments directly revealed the crosslinking degree of the P-*a*POPs under different reaction conditions (Fig. 18c, d)^39,58,95^. The higher of the reaction rates, the more uniform of crosslinking P-centers.

**Fig. 19:**
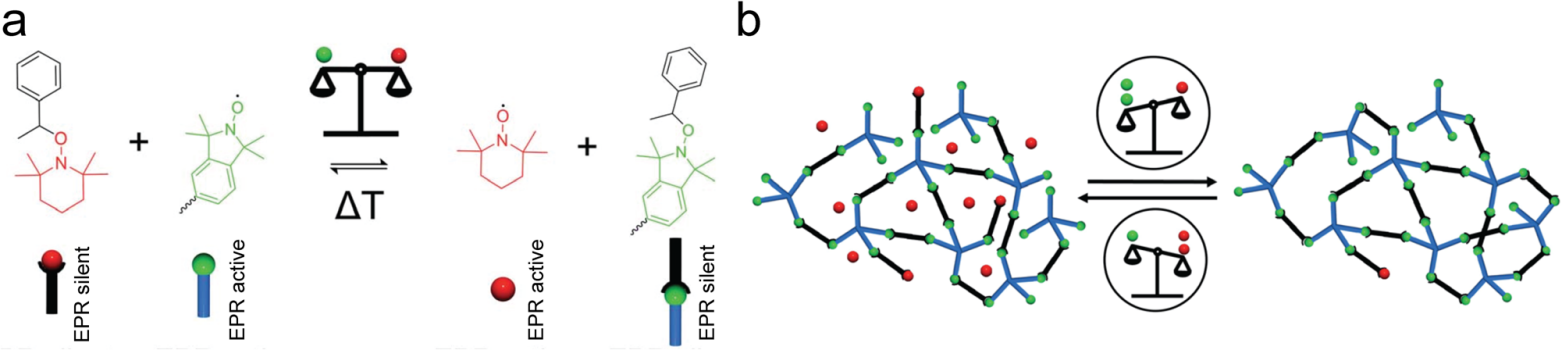
Solid state electron paramagnetic resonance studies for P-aPOPs containing various P-environments. **a** Dynamic equilibrium in the nitroxide exhange reaction using two different nitroxides (red: TEPO. green: isoindoline). **b** Tuning of crosslinking degree via equilibrium control in the nitroxide exchange reaction. (adapted with permission from ref. ^123^ © 2023 Royal Society of Chemistry).

In addition to the above solid state NMR technique, Tsotsalas and coworkers also explored the use of solid state electron paramagnetic resonance spectroscopy technique to quantify the crosslinking degree in N-*a*POPs (Fig. 19)^123^. The nitroxide exchange reaction allowed them to not only modify the EPR active crosslinking TEMPO nitroxide center, but also reveal the impacts of the crosslinking center on the porosity of the materials.

### Challenges and future directions for MG-Amorphous POPs

As a subgroup of *a*POPs, the unique family of MG-*a*POPs just started its journy recently. Although the rich chemical and electronic configurations of main group elements successully endowed the *a*POPs with the diverse chemical structures, properties, and functions in the unprecedent areas, there are still lots of challenges from the prospective of the chemical-structure and solid-state-structure probing, the porous-structure tuning, and the thin-film state developing.

Solid state NMR/FTIR experiments were widely used to probe the chemical structures of MG-*a*POPs in previous studies. Due to the limited sensitivity and resolution, these experiments hardly provided the full and precise chemical information of MG-*a*POPs. According to the polymer digestion method designed by Bunz and coworkers, the chemical compositions of MG-*a*POPs may not always be the same as the small molecule reactions^78^. Therefore, for any newly synthesized MG-*a*POPs, multiple chemical characterization methods, such as solid state FTIR/NMR, EA, ICP-OES, XPS, etc., are strongly suggested to apply, which can provide the more detailed and reliable chemical information. Even though, under some specific situations, as such in full carbon-based *a*POPs, the full and precise chemical information is still difficult  to obtain due to the very similar chemical environments of the carbon elements within the reaction mixtures. Therefore, new experimental techniques with high sensitivity and resolution are exceedingly desirable for the developments of MG-*a*POPs in the future.

Different from *c*POPs where the solid-state structures can be successfully probed by the X-ray diffraction experiments, the solid-state stucture information of MG-*a*POPs is hard to uncover due to the lack of long-range order. Previous studies showed that porous structures of *a*POPs is not crystallographically defined by the monomer structures as observed in the crysalline porous materials^8,30^. It is generally believed that the porosity is due to less efficient struct packings in the amorphous polymeric networks, in which the average pore sized distribution envelop is related statistically to the stuctures of monomers (average length, conformational freedom and topological geometries) and the interpenetration of polymer chains. We believe that the same situation is also applicable to MG-*a*POPs in term of porosity formation.

As discussed in the previous section, few of the classical materials characterization techniques were able to reveal the solid-state structure information of MG-*a*POPs. Although the solid-structures cannot be fully revealed, the solid-state structure information of MG-*a*POPs, such as porous environments and crosslinking environments, was able to be quanitatively uncovered by using solid state NMR experiments in the recent studies^58^. The solid state NMR experiments harnessed a new strategy to probe the environments of crosslinking centers, in which NMR active main group elements (such as B and P) were used as the direct crosslinking centers. In the future, it is believed that polymerizations having other NMR active main group elements (such as Si, Al, Ga, etc.) as the direct crosslinking points will also allow the solid state NMR experiments to directly probe the chemical environments of these crosslinking centers.

With more reliable information on the crosslinking environments, more insightful information on the relationship between the chemical structures, porosity characteristics, and functions was able to be obtained. Therefore, the design of new polymerization chemistry involving NMR active main group elements and carbon bond formation could be one promising area for the better controlled chemical and porous stuctures of *a*POPs in the future. Particularly, a great number of heavier main group elements will be also suitable for constructing MG-*a*POPs when choosing appropriate synthetic chemistry. The different synthetic protocols, oxidation state, and coordination chemistry of heavier main group elements will lead to new horizons in the field. For example, compared with trivalent B-center, Al-center may endow polymeric structures with enhanced Lewis acid characteristics and tunable topological structures where Al-center can adopt trigonal planar, tetrahedral, and trigonal bipyramid structures.

Along with probing the solid structures, controlling the chemical and porous structures is another challenge that limited the advance of MG-*a*POPs. The irreversible chemical bond formation character of *a*POPs intrinsically constrained the control of porous structure formation. Altough constructing MG-*a*POPs with uniform microporosity, even ultramicroporosity are not impossible in the recent studies^8,58,83–85,87,97^, the fundemental principles for achieving the uniform microporosity and ultramicroporosity are yet to be uncovered and generallized. Recent studies clearly showed that reaction conditions, such as monomer ratios, catalysts, ligands, and solvents, have strong impacts on the chemical structures and porous structures of *a*POPs synthesized by classical transistion metal catalyzed coupling reactions^1–3,8,38,58,78,87,113–116,122^. For example, the same monomers afforded the very different porous structures under the different polymerization conditions, such as reaction rates^58,115^. Therefore, exerting more efforts on revealing the fundamental principles is expected as the new reserch directions for MG-*a*POPs in the future.

The insoluble character of *a*POPs also limitd their potential applications in some specific areas, such as organic electronic devices where materials in the thin-film state are generally required. Recent progress showed that the in-situ surface electropolymerization method can be nicely harnessed to construct the thin-film state of B-*a*POPs that maintain good porosity characters and better surface morphologies^73^. Furthermore, the thin film of MG-*a*POPs with large surface areas and uniform surface morphologies are expected to find various applications of electronic devices, photocatalysis, and toxic gases sensing. However, the in-situ surface electro-polymerization strategy has not been fully recognized in the field of *a*POPs. Recent bloom of electrochemical organic synthesis involved main group elements and carbon bond formations potentially provide new opportunities for constructing MG-*a*POP based thin films as the new research direction in the future^124^.

### Conclusion and outlook

As a subgroup of *a*POPs, MG-*a*POPs indeed witnessed the fast development in recent years. This review highlighted some distinct examples of MG-*a*POPs containing Group-13, Group-14, Group-15, and Group-16 elements. The unique chemical geometries and electronic configurations of main group elements played important roles in tuning the chemical structures, porosities, and properties of the MG-*a*POPs. Topological structures of the various main group element showed the strong impacts on the porous characteristics of MG-*a*POPs. Particularly, the rigid 3D main-group centers endowed MG-*a*POPs with the significantly high surface areas. The electron-rich and electron-poor characters of main group element fruther modulated the redox, optical and electronic properties of the MG-*a*POPs in another dimension. The review also discussed the new main group element chemistry applied for synthesizing MG-*a*POPs. These studies will catalyze more new synthetic protocols involving unexplored main group element chemistry for designing new functional MG-*a*POPs in the future.

Later, the review further covered the main characterization techniques designed for MG-*a*POPs. The amorphous nature of MG-*a*POPs made the structure elucidation very challenging. Using the unique main group elements as the direct crosslinking centers may offer an alternative method to shed light on the relationships between crosslinking environments, porosities and properties. It was anticipated that the uncovered insights can provide guidance, and enlighten future research efforts in advancing the field. Bridging rich main-group chemistry and the porous structure is clearly appealing. More unexpected and exciting findings on the structures and properties of MG-*a*POPs are to be discovered in the emerging field through the merry marriage.
